# A scaling-free minimum enclosing ball method to detect differentially expressed genes for RNA-seq data

**DOI:** 10.1186/s12864-021-07790-0

**Published:** 2021-06-26

**Authors:** Yan Zhou, Bin Yang, Junhui Wang, Jiadi Zhu, Guoliang Tian

**Affiliations:** 1 College of Mathematics and Statistics, Institute of Statistical Sciences, Shenzhen Key Laboratory of Advanced Machine Learning and Applications, Shenzhen University, Shenzhen, China; 2 School of Data Science, City University of Hong Kong, Hong Kong; 3 Department of Statistics and Data Science, Southern University of Science and Technology, Shenzhen, China

**Keywords:** Minimum enclosing ball, Differentially expressed genes, RNA-seq data

## Abstract

**Background:**

Identifying differentially expressed genes between the same or different species is an urgent demand for biological and medical research. For RNA-seq data, systematic technical effects and different sequencing depths are usually encountered when conducting experiments. Normalization is regarded as an essential step in the discovery of biologically important changes in expression. The present methods usually involve normalization of the data with a scaling factor, followed by detection of significant genes. However, more than one scaling factor may exist because of the complexity of real data. Consequently, methods that normalize data by a single scaling factor may deliver suboptimal performance or may not even work.The development of modern machine learning techniques has provided a new perspective regarding discrimination between differentially expressed (DE) and non-DE genes. However, in reality, the non-DE genes comprise only a small set and may contain housekeeping genes (in same species) or conserved orthologous genes (in different species). Therefore, the process of detecting DE genes can be formulated as a one-class classification problem, where only non-DE genes are observed, while DE genes are completely absent from the training data.

**Results:**

In this study, we transform the problem to an outlier detection problem by treating DE genes as outliers, and we propose a scaling-free minimum enclosing ball (SFMEB) method to construct a smallest possible ball to contain the known non-DE genes in a feature space. The genes outside the minimum enclosing ball can then be naturally considered to be DE genes. Compared with the existing methods, the proposed SFMEB method does not require data normalization, which is particularly attractive when the RNA-seq data include more than one scaling factor. Furthermore, the SFMEB method could be easily extended to different species without normalization.

**Conclusions:**

Simulation studies demonstrate that the SFMEB method works well in a wide range of settings, especially when the data are heterogeneous or biological replicates. Analysis of the real data also supports the conclusion that the SFMEB method outperforms other existing competitors. The R package of the proposed method is available at https://bioconductor.org/packages/MEB.

**Supplementary Information:**

The online version contains supplementary material available at (10.1186/s12864-021-07790-0).

## Background

Next-generation sequencing (NGS) provides an attractive alternative for quantitative analysis of the underlying complexities of gene expression [1]. The affordability and effectiveness of high throughput sequencing has led to its rapid application in a wide range of biological and medical research. In particular, RNA sequencing, which involves the mapping of sequenced fragments of cDNA to a reference genome or transcriptome, has been used for genotyping analysis, detection of methylation patterns [2], and identification of transcription factor binding sites [3]. In RNA-seq experiments, the number of sequenced fragments mapped to a feature is used to measure the expression level of a feature [4]. However, using RNA-seq data for the detection of differential gene expression under different biological conditions or in different species remains a challenge for practitioners.

Recently, several statistical methods have been developed to identify differentially expressed genes in the same or different species. For the same species, the existing methods broadly fall into two categories. The first one is parametric methods, such as edgeR [5] and DESeq [6], which assume that RNA-seq data follow a negative binomial distribution, and HTN [7], which assumes a Poisson distribution for the read counts. The second one is non-parametric methods, such as NOIseq [8] and LFCseq [9]. For different species, Brawand et al. [10] proposed a method based on reads per kilobase of transcript per million mapped reads (RPKM) [11] to employ 1000 of the most conserved genes to obtain the median expression level, and they regarded the ratio of the median values as a scaling factor. Zhou et al. [12] considered different structures between the same and different species, and proposed a scale-based normalization (SCBN) method that utilizes a part of the known conserved genes and the hypothesis testing framework.

Notably, normalization is essential to all these methods, and Evans et al. [13] classified the existing normalization into three categories. Total count [14] and RPKM assume that two samples have the same number of total short reads, and RNA-seq data normalization is based on library size. DESeq and trimmed mean of M-values (TMM) [15] normalize the RNA-seq data by distribution or testing whether differentially expressed (DE) and non-DE genes in the dataset have the same or balanced expression. In addition, some methods perform the normalization using control genes, like housekeeping genes [16] or spike-ins [17]. Note that the gene number and sequencing depth are natural factors that are considered in normalization for the same species. However, we also need to consider data constituents and gene lengths corresponding to orthologous genes in different species. Normalization is so essential for differential expression analysis that Evans et al. [13] thought that errors in normalization could have a severe influence and even mislead the downstream analysis.

In this paper, we propose a method that extends the minimum enclosing ball (MEB) method in machine learning and is able to detect DE genes. Because the proposed method is only based on the similarity of the non-DE genes and differences between the non-DE and DE genes, which does not require the normalization step with a scaling factor, so we called it scaling-free minimum enclosing ball (SFMEB) method. The MEB method was first proposed by Elzinga et al.[18] to find the smallest ball to enclose all the given sample points. In this way, it is similar to the support vector data description [19] designed to detect novelties or outliers. This property enables SFMEB to identify the homologous points in the enclosing ball and to detect points that differ substantially from the points in the ball. Specifically, SFMEB constructs a spherically shaped decision boundary for the enclosed sample points, and a point will be discriminated as an outlier if it lies outside the ball. Similar to the support vector machine [20], the sample points are implicitly mapped into high-dimensional feature spaces by a non-linear transformation, so that kernel tricks can then be utilized to increase the flexibility of the SFMEB method. In the model, the house-keeping genes are only used as a part of non-DE genes, and other non-DE genes would be similar to the house-keeping genes. These similar non-DE genes can be catched by the SFMEB model through a compact enclosing hypersphere in the feature space, while the DE genes, assumed to be different from the non-DE genes, are very likely to be outside the enclosing ball.

We have extended the SFMEB method to detect DE genes by first assuming knowledge of a small set of housekeeping genes (in same species) or conserved genes (in different species). We then construct a SFMEB to enclose as many as possible of these non-DE genes without substantially enlarging the size of the SFMEB. We control type I errors by setting the proportion of the non-DE genes which are outside of the SFMEB and thus regarded as DE genes. The computation is expedited, as suggested by Tsang et al. [21], by using core vector machines (CVM) as one constant kernel function and extending it to the general core vector machines [22] to work for any kernel function. Hu et al. [23] also proposed a fast learning algorithm (FL-TMEB) for SFMEB with a soft margin. In the present paper, we considered the size of the real data and we used the algorithm proposed by Chang et al. [24] to facilitate the computation.

The real data we analysed involve the same species under different conditions, as well as different species. For the same species, the data consist of RNA-seq short read counts from the liver and kidney, including 5 biological replicates for each sample (see Marioni et al. [25] for details). Human housekeeping genes were those used previously [26] (see Eisenberg et al. [27] for a description of these genes). By excluding the obsolete transcripts, which can be inquired in the NCBI database [28], we eventually obtained 16519 genes and 530 housekeeping genes for the subsequent analysis. For different species, real data were obtained from Brawand et al. [10], which includes two groups of orthologous transcripts of humans and mice. Following the pre-process steps described by Zhou et al. [12], we obtained 19330 available orthologous transcripts, including 143 most conserved orthologous transcripts.

**Fig. 1:**
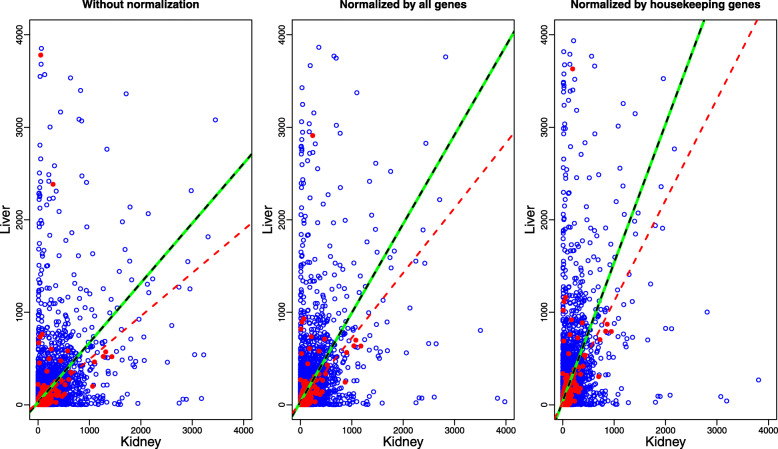
The scatter plots and the fitted linear model of the short read counts of genes from a library of liver and kidney [25]. Each point represents a gene, and the horizontal and vertical coordinates represent the gene counts in liver and kidney. Red points represent the housekeeping genes, and blue points represent the remaining genes. The red dash lines are fit on the housekeeping genes, the black dash lines are fit on the remaining genes, and the green solid lines are fit on all genes

Figure 1 shows the scatter plots of the short read counts of genes in a random sample from a library of liver and kidney [25] and the corresponding fitted linear models in three cases. Only those genes with count number no greater than 4000 are displayed in Fig. 1. In Fig. 1, the left panel directly fits a linear model on the original data, the middle panel fits a linear model with the normalized data, where the original data are normalized by a scaling factor which is estimated by TMM method [15] for all genes, and the right panel also fits a linear model on the normalized data, where the data are normalized by a scaling factor estimated by TMM method with housekeeping genes. Table 1 shows the coefficients of the fitted lines for these three cases. It is clear that the slopes of fitted lines of housekeeping genes in the first two cases are less than 1, which suggests that the original data or normalized data could not present the true expressions of genes. And in the last case, the slope of the fitted line of housekeeping genes is a little more than 1, which may be caused by a few housekeeping genes that are much higher than the red dash line. Thus, there may be more than one scaling factors in this real dataset, and normalization with two or more scaling factors can be much more complex or even unprocurable.

**Table 1:** The estimated coefficients of fitted linear models of real data of liver and kidney

		Intercept	Coefficients
Non-normalized	Housekeeping genes	24.6171	0.4668
	Remaining genes	26.1319	0.6457
	All genes	25.7412	0.6453
Normalized	Housekeeping genes	21.0627	0.7025
by all genes	Remaining genes	32.1974	0.9629
	All genes	31.4334	0.9622
Normalized	Housekeeping genes	26.273	1.093
by housekeeping genes	Remaining genes	40.163	1.498
	All genes	39.210	1.497

Supplementary Figures S1 and S2 show the distributions of the absolute log-fold changes (base 2) for the same and different species. If we regard the transcripts with a log ratio of expression level (after normalization) less than 1 as non-DE, we can see that most of the transcripts are non-DE. However, some log ratios can be much greater than 1, even approaching 6, suggesting that those transcripts have relatively higher expression levels. In this paper, the dataset with clearly different log-fold-changes in different parts of the genes is recognized as heterogeneous data. The distribution of the same or different species shows that heterogeneity is very common in real data. In addition, in real data, over-dispersion is also very common in biological replicates; that is, the variance of biological replicates is uncontrollable. The use of traditional methods that depend on a single scaling factor is no longer feasible with real data, and this motivated us to develop a method for detection of DE genes without normalization.

## Results

In this paper, we proposed SFMEB method to detect DE genes. The details of the SFMEB model have been shown in “Methods” section. To validate the performance of proposed method, we consider simulation studies and real data analysis with same and different species. All the R scripts that analysed the data have been uploaded at github, which could be accessible at https://github.com/FocusPaka/NIMEB.

### Simulation studies

We evaluate the performance of the proposed method by conducting simulations in a variety of scenarios and comparing the results with those obtained with five existing methods using the criteria of receiver operating characteristic (ROC) curve and area under the curve (AUC). The five existing methods including Library Size, edgeR [5], DESeq [6], HTN [7], and NOISeq [8], which are widely used in the literature of DE gene expression.

#### Simulation design

Let *G* be all genes, and *j* (*j*∈{1,2}) be the condition, *j*_*r*_ be the *r*-th replicate of the *j*-th condition. Then $x_{ij_{r}}$ represents the observed number of short reads of gene *i* in sample *j*_*r*_. In order to mimic real data, the simulation data for the same species includes both non-DE genes and DE genes. The DE genes have two scenarios: one is genes with multiple expression levels and the other is genes only expressed in one condition. We first set the proportion of DE genes with multiple expression levels in all genes (not including uniquely expressed genes) as *π*_0_ with *d* log-fold changes (base 2). Of those DE genes, the proportion of *p*_*j*_ are randomly up-regulated under condition *j*. In addition, the uniquely expressed genes for two conditions are denoted as a vector *u*. We then generate the simulation data following the steps of Robinson and Oshlack (2010) [15]. Considering the complexity of real data, we also use an RNA-seq data simulater (compcodeR::generateSyntheticData(), Soneson and Delorenzi (2013) [29]) to generate the counts for each gene. The simulation data are sampled from a negative binomial (NB) distribution. Both methods are achieved by R codes, the former provides R scripts in [15], the latter is carried out by function ‘generateSyntheticData()’ which is included in R package ‘compcodeR’. In our work, the simulation data are RNA-seq counts in each gene of different samples. We conduct the differential expression analysis with the counts matrix. Generally, we do not directly analyse the differential expression of RNA-seq reads, which are obtained in the sequencing experiment. The reads are usually mapped to the reference genome, and be transformed to counts matrix for the downstream analysis. Therefore, all datasets in our simulation studies are counts data.

The first four simulation studies are conducted following the steps of Robinson and Oshlack (2010) [15]. We consider the simulation data either with or without biological replicates, and with or without heterogeneity. Note that edgeR, HTN, DESeq and NOISeq need to normalize data beforehand, whereas Library Size directly tests the data without normalization. For the last two simulation studies, the simulation data are generated by the function of generateSyntheticData() in R package ‘compcodeR’ [29]. In these two studies, we consider the simulation data with or without heterogeneity. The six different simulation designs are summarized in Table 2.

**Table 2:** Simulation designs for the Studies 1-6

Simulation methods		Non-heterogeneity	Heterogeneity	Methods
	Without	Study 1	Study 2	SFMEB,
Robinson and Oshlack (2010) [15]	replicates			Library Size,
	With	Study 3	Study 4	edgeR,
	replicates			HTN,
Soneson and Delorenzi (2013) [29]	With	Study 5	Study 6	DESeq,
	replicates			NOISeq

We first consider the non-heterogeneous data without replicates in Study 1. The simulation data have only one scaling factor, and each condition includes only one sample as the non-heterogeneous data without replicates. The parameters are set as follows. In this case, excluding the uniquely expressed genes, we generate 15,000 genes for the two conditions, and the proportion of DE genes *π*_0_ changes from 0.3 to 0.7 by steps of 0.2. Of those DE genes, 90% show 2 log-fold (base 2) up-regulation in the second condition. To mimic real data, we analysed two real datasets which have been introduced in “Background” section, and calculated the number of possibly uniquely expressed genes in different organs or species in Supplementary Table S1. For the same species, the RNA-seq short counts are from the liver and kidney [25], which including 5 biological replicates for each sample. There are 508 unique genes in kidney and 1099 unique genes in liver, which may affect the result of the model. For the different species, real data were obtained from Brawand et al. (2011) [10], which includes two groups of orthologous transcripts of humans and mice. There also exist 833 unique genes in human and 987 unique genes in mouse. It is clear that the numbers of possibly uniquely expressed genes in each sample are about 3*%*∼6*%*. Thus, in this simulation study, we set uniquely expressed genes for two conditions as *u*=(1000,500).

Unlike in Study 1, we consider heterogeneous data with two scaling factors in Study 2. We obtain the heterogeneous dataset by generating two groups of data in the same way, and we then combine them into one dataset. The first dataset includes 15,000 genes. Of these, 60% are DE genes at the 2 log-fold level. Of that 60%, 90% are up-regulated in condition 2. The uniquely expressed gene number is *u*=(1000,500). The second dataset includes 10,000 genes, and we gradually changed the proportion of DE genes from 0.1 to 0.5 by steps of 0.2. This revealed that 10% of the DE genes showed a 3 log-fold upregulation in condition 2. The number of uniquely expressed genes for the two conditions in the second data is *u*=(800,1500).

In Study 3, we test the performance of all the methods for the non-heterogeneous data with biological replicates. We fix *G*=15,000,*π*_0_=0.6,*p*_2_=1,*d*=2 and *u*=(1000,800), and we consider the case in which the number of biological replicates under the two conditions changes from 2 to 8 by step 3.

The forth study considers the heterogeneous data with replicates. Following the same steps as in Study 2, we also have two datasets and equal numbers of biological replicates in Study 4. In the first dataset, we have *G*=15,000,*π*_0_=0.6,*p*_2_=0.9,*d*=2, and *u*=(1000,800). In the second dataset, the parameters are set as *G*=10,000,*π*_0_=0.4,*p*_2_=0.1,*d*=3, and *u*=(2000,1000). The number of biological replicates varies from 2 to 8 by step 3.

In the last two studies 5 and 6, data are generated by an RNA-seq data simulator (compcodeR::generateSyntheticData()). Similarly, in Study 5, we consider the non-heterogeneous data with biological replicates. The simulation data are generated from negative binomial (NB) distribution with varied logFCs. In Study 6, we generate the heterogeneous data with replicates. The data are obtained by two combined datasets sampled from NB distribution.

In Study 5, we generate 15,000 genes in two conditions, and each condition has two samples. There are 60% DE genes at the no less than 3 log-fold level, and all the DE genes are up-regulated in condition 2. The number of biological replicates varies from 2 to 8 by step 3. We consider the dataset with two scaling factors in Study 6. The parameters are set as follows: in the first dataset, we have 15,000 genes, and 60% are DE genes at the no less than 2 log-fold level. All the DE genes are up-regulated in condition 2. In the second dataset, we have 10,000 genes, and 40% are DE genes at the no less than 3 log-fold level, of which 10% are up-regulated in condition 2. Likewise, the number of biological replicates varies from 2 to 8 by step 3.

#### Evaluation criteria

We assess the performance of the proposed SFMEB method using the area under a receiver operating characteristic (ROC) curve (AUC). Note that many popular criteria, like FDR, Precision, Sensitivity and Specificity, are strongly influenced by the choice of thresholding values.

The significance level for differential expression between conditions is used to rank the genes from the most to the least significant. We then plot the receiver operating characteristic (ROC) curve, where the horizontal ordinate is the false positive rate (FPR) and the vertical coordinate is the true positive rate (TPR). The area under the ROC curve is computed as the AUC, and a larger AUC value indicates the overall discriminating ability. For SFMEB, we use the distance between the point to the decision hypersphere in feature space to measure the significance level of a gene being a DE gene. The distance can be positive or negative, depending on whether it is outside the hypersphere or not. Thus we can rank the genes based on the signed distance, and the larger the distance is, the more possible is the gene a DE gene. For Library Size, edgeR, HTN and DESeq, we use estimated p-values for ranking. For NOISeq, the genes are ranked by the estimated probabilities of the genes being DE genes.

#### Simulation results

**Fig. 2:**
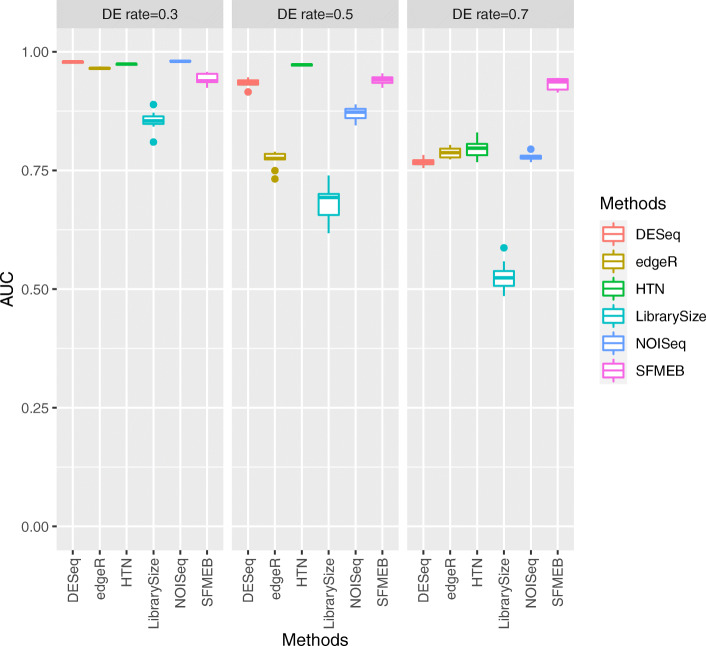
In Study 1, the simulation data are non-heterogeneous without replicates. The AUC values of six methods are shown when the proportion of DE genes varies from 0.3 to 0.7 by steps of 0.2

**Fig. 3:**
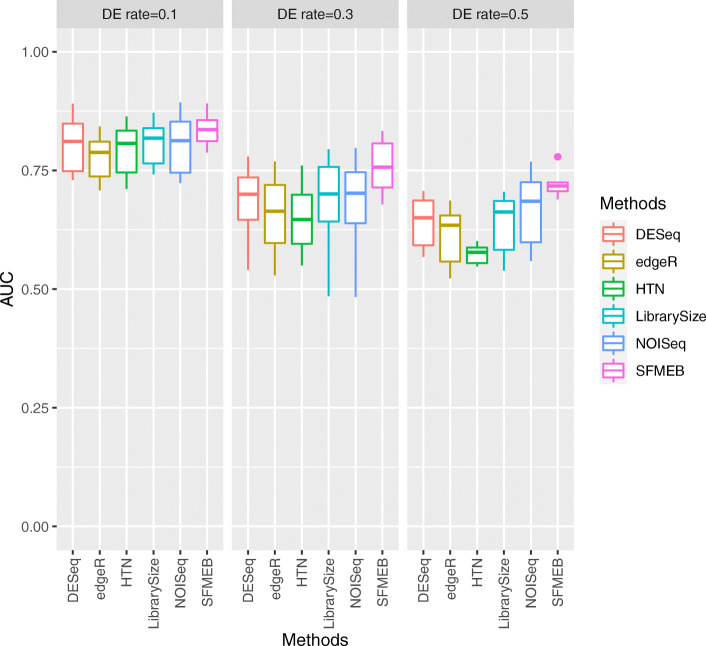
In Study 2, the simulation data are heterogeneous, with two scaling factors, and without biological replicates. The proportion of DE genes in one of the datasets is fixed at 0.6, and the AUC values of six methods are shown when the proportion of DE genes in another datasets changes to 0.1, 0.3, or 0.5

**Fig. 4:**
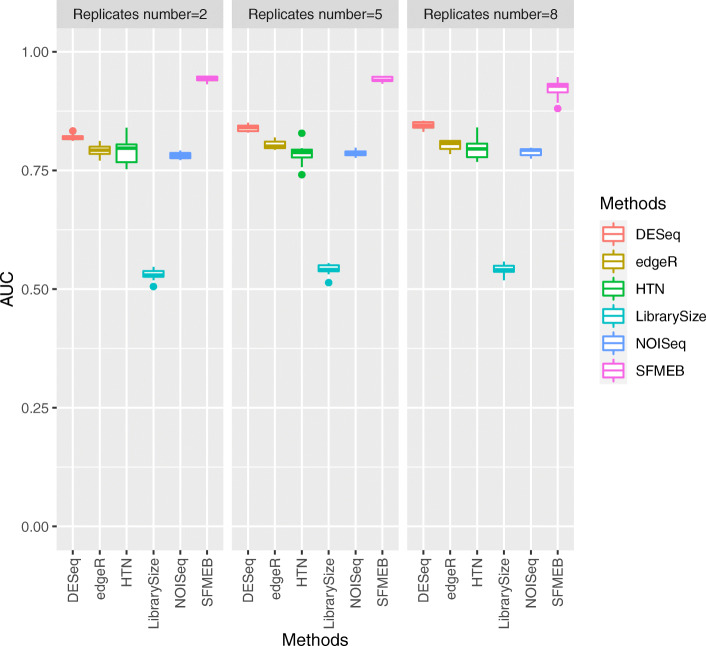
In Study 3, the simulation data are non-heterogeneous with replicates. The AUC values of six methods are shown under the different number of biological replicates

**Fig. 5:**
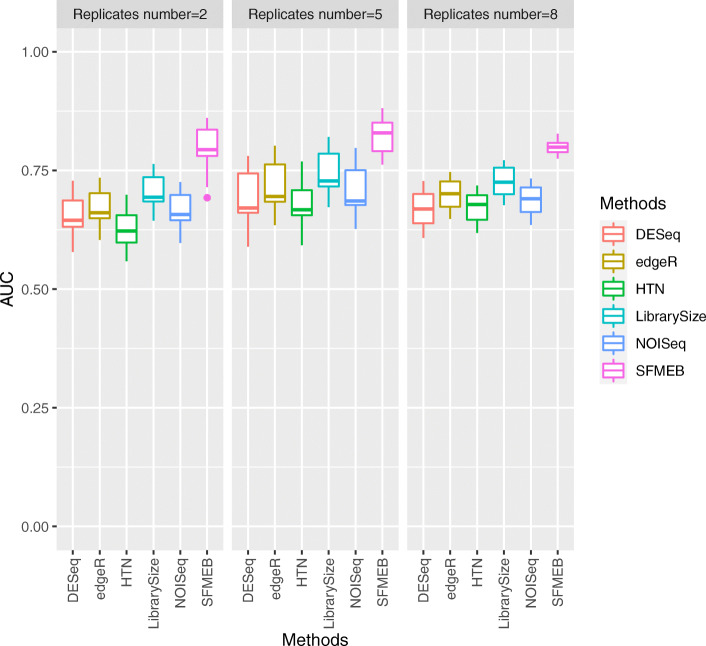
In Study 4, the simulation data are heterogeneous, with two scaling factors, and with replicates. The AUC values of the six methods are shown when the number of biological replicates equals to 2, 5, and 8

**Fig. 6:**
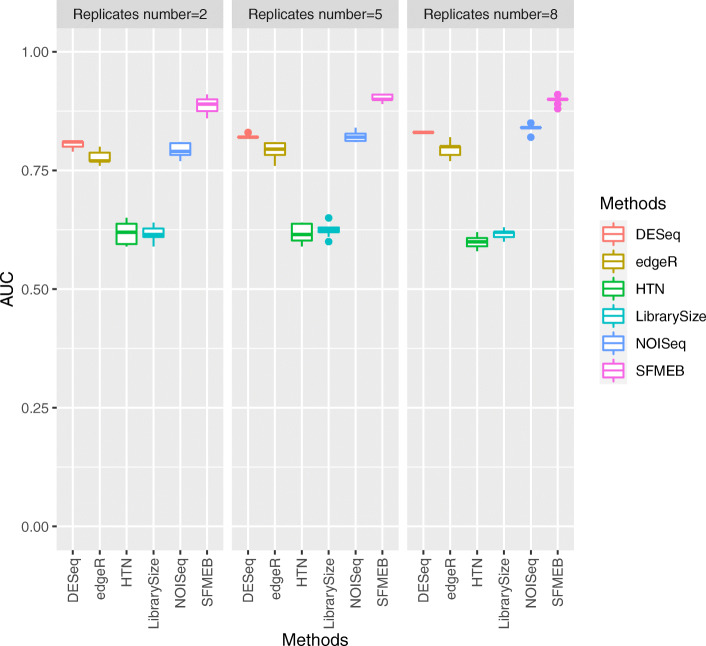
In Study 5, the simulation data are non-heterogeneous with biological replicates, and data are generated by an RNA-seq simulator (compcodeR::generateSyntheticData(), Soneson et al. (2013)). The AUC values of the six methods are shown under the different number of biological replicates

Each simulation study is repeated 20 times, and the averaged performance for six methods, along with various parameters, are reported in Figs. 2, 3, 4, 5 and 6. More simulation results can be found in Supplementary FiguresS3-S10. Simulation data for Supplementary Figures S3-S6 are generated by the method in Robinson and Oshlack (2010) [15]. S3-S4 for non-heterogeneous data without biological replicates, in Supplementary Figures S5-S6 for heterogeneous data without biological replicates. Simulation data for S7 are generated by an RNA-seq data simulator (compcodeR::generateSyntheticData()). In S8-S10, we compare the overall performance of six methods under six studies.

Study 1 investigates the ROC and AUC performances for non-heterogeneous data without replicates. Figure 2 plots the AUC values of six methods when the proportion of DE genes varies from 0.3 to 0.7 by steps of 0.2. SFMEB method shows its superiority over other methods when the proportion is over 50%. Supplementary FiguresS3 and S4 show the discrimination results for six methods. Each point in the plot represents a gene, the coordinates of point are the counts of gene in two samples. For clarity, we only display the genes whose read counts are no greater than 600. When the proportion of DE genes is small, DESeq and NOISeq perform better for the detection of true negative genes, but they yield higher false negatives when the proportion is 0.6. Therefore, SFMEB outperforms other methods when the proportion of DE genes is large.

Study 2 investigates the heterogeneous data including two scaling factors without biological replicates. Figure 3 shows the AUC values of the six methods when the proportion of DE genes in one of the datasets changes to 0.1, 0.3, and 0.5. The figure shows that the performance is better with SFMEB than with the other methods in this case. Supplementary Figures S4 and S5 show the discrimination results. The performance of Library Size is generally unsatisfactory, as DESeq yields a large number of false negative genes, edgeR and HTN become ineffective at detecting genes when the number of false positives increases and NOISeq performs better with a smaller proportion of DE genes. Likewise, SFMEB performs the best with heterogeneous data when the proportion of DE genes is large.

We also mimic the real scenarios by generating data with biological replicates in Studies 3 and 4. We analyse non-heterogeneous data with replicates in Study 3. Figure 4 shows that the AUC values are much larger for SFMEB than for the other methods under different number of biological replicates. In summary, SFMEB is a good method for handling data with biological replicates. Study 4 considers heterogeneous data including two scaling factors with biological replicates. Figure 5 shows the AUC boxplots for six methods when the number of biological replicates equals to 2, 5, and 8. The AUC performance of SFMEB confirms the same conclusions with Study 3.

The simulaiton data analysed in Studies 5 and 6 are generated by an RNA-seq simulator (compcodeR::generateSyntheticData(), Soneson et al. [29]). In Study 5, we consider to generate the non-heterogeneous data with biological replicates by the new RNA-seq simulator. Figure 6 shows the AUC values of six methods for different number of biological replicates in each condition. The six methods have a stable performance in this case, and SFMEB shows its superiority over other methods for different number of biological replicates. In Study 6, we consider the heterogeneous data including two scaling factors with biological replicates that generated by a NB distribution. Similarly, we compare the performance of six methods for the different number of biological replicates, and the results are shown in the SupplementaryFigures S7. We also find that SFMEB performs better than the other methods for different number of replicates.

In each simulation study, we compare the performance of six methods for the different logFC values and the different proportions of up-regulated in all DE genes. Sup-plementary Figure S8 shows that the ROC curves of six methods when simulation data are non-heterogeneous and without replicates (Study 1), and under different logFCs and different up-regulated proportions. In this case, SFMEB performs better for a higher logFC or a higher proportion of up-regulated in all DE genes. Besides, SFMEB has a robust performance for varied logFCs and up-regulated proportions. In SupplementaryFigure S9, we compared that for six different simulation datasets, the performance of six methods when the value of logFCs equals to 1, 2, and 3. Panels A to F are corresponding to the six studies in the simulation studies. The data in panels A to D are generated by a Poisson distribution [15], and the data in panels E and F are generated by a negative binomial distribution [29]. In summary, compared with the other five methods, SFMEB performs more robust with varied logFC values, and outperforms other methods with larger logFC values. Especially, SFMEB method shows its superiority over other methods for two scaling factors data. SupplementaryFigure S10 shows the AUC performances of in six studies under different proportion of up-regulated genes. For the case of 50:50 odds, SFMEB performs a little worse than the other methods for one scaling factor. For the case of 70:30 odds, SFMEB is comparable with the best performance HTN method for one scaling factor. However, our proposed method is much better than the other methods for the case of 90:10 odds. For two scaling factors, the SFMEB method outperforms the other methods with different ratios of up-regulated genes. In total, the proposed method has a robust performance in a balance proportion of up-regulated genes and outperforms other methods with a higher proportion of up-regulated genes in one condition. Note that, the data with a higher proportion of up-regulated genes in one condition or with a higher value of logFC will inflate the library size of the up-regulated group, which has a great impact on the performance of library-based methods, leading to poorer results for methods that normalise based on library size.

#### Simulation with different species

We used the same simulation setup describe by Robinson et al. [15] to generate a dataset for two species. We compared SFMEB with Median [10] and SCBN [12]. Note that, except for DE and non-DE orthologous genes, we also include unmapped genes, which represent genes that only exist in one of the species; these are denoted by a vector **s**. Here, we generate two groups of datasets by only considering heterogeneous data including two scaling factors in the different species. In the first dataset, let *G*=12,000,*π*_0_=0.4,*p*_2_=0.1,*d*=3,*u*=(1000,1200), and *s*=(1000,800). In the second dataset, let *G*=10,000,*p*_2_=0.9,*d*=2,*u*=(2000,1000), and *s*=(3000,2000). The proportion of DE orthologous genes changes from 0.3 to 0.7 by step 0.2.

Supplementary Figure S11 displays the AUC values of three methods when the proportion of DE orthologous genes equals to 0.3, 0.5, and 0.7. Compared with other two methods, SFMEB has a relatively higher AUC value. The discrimination result in Supplementary Figure S12 shows that Median and SCBN have more false positive genes; however, SFMEB has less false positive genes and more false negative genes. Therefore, SFMEB performs best at precise discrimination between DE and non-DE genes.

### Real data analysis

In this section, we apply the proposed SFMEB method to analyse a liver and kidney [25] dataset for the same species and human and mouse [10] dataset for different species. We compare its performance with other existing methods.

For the liver and kidney [25] RNA-seq count data, there are two tissues of a male, each of which includes 5 biological replicates. The RNA-seq short reads are sequenced by Illumina sequencing platform and then mapped to the reference genes. We consider that 10% of the housekeeping genes are DE genes, so we finally obtain 1681 DE genes with the SFMEB method. For Library Size, edgeR, HTN and DESeq, we also regard the 1681 genes with the smallest p-values as DE genes. For NOISeq, the 1681 genes with the largest probabilities are considered DE genes. We then use these 1681 DE genes detected by each method to report the number of false detected housekeeping genes. In addition, we report the number of false detected in the 500 and 1000 most significant DE genes. Comparison with the other five methods in Table 3 confirms that SFMEB detects the smallest number of housekeeping genes in the detected DE genes in all three cases, leading to the smallest FDR. Note that SFMEB performs better than other methods with the criteria, because it builds the model by identifying outliers compared to housekeeping genes. There may be a biases comparison since we have no ground truth in real data analysis. Whatever, one of the key advantages of SFMEB is that one can control the type I error and ensure a lower FDR.

**Table 3:** The number of detected housekeeping genes in the most significant 500, 1000, and 1681 DE genes for the six methods

Number of DE genes	500	1000	1681
SFMEB	6	13	43
Library Size	17	33	52
edgeR	13	27	54
HTN	13	27	54
DESeq	12	30	55
NOISeq	14	28	49

Next, we consider the computational cost of six methods for DE gene detection. Supplementary Table S2 shows the CPU time (seconds) of all methods for different sample sizes in the real data of liver and kidney [25]. Compared with other methods, the SFMEB method spends less time, especially for larger sample size. HTN will spend much more time than the other methods, which may be caused by the process of calculating scaling factor for each sample to the reference sample.

**Fig. 7:**
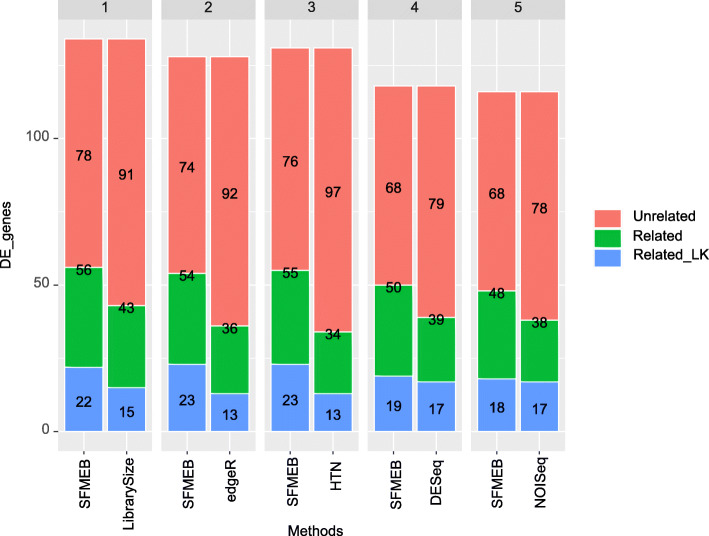
The pairwise comparison between SFMEB and the other five methods for the 200 most significant DE genes, excluding common genes. The barplots show the number of genes related to illness, liver and kidney (Related), the number of genes related to liver and kidney (Related_LK), and the number of genes unrelated to illness, liver or kidney (Unrelated)

Finally, we assess the biological function of the detected DE genes to compare the accuracy of six methods. We first take pairwise comparisons for the six methods. The results of overlapping genes are shown in Supplemen-tary Figure S13. Note that SFMEB detects less common genes compared with the other methods. In fact, SFMEB employs a completely different method for detecting DE genes, and this also enables us to find different significant DE genes. In this paper, we consider the comparison between SFMEB and the other methods, as shown in the first line of Supplementary Figure S13. We exclude the overlapping genes and analyse the rest of genes detected by each method as the 200 most significant genes to determine how many are related to illness, liver and kidney, and how many are related to liver or kidney. The biological function of each gene could be inquired at the NCBI website [28]. The pairwise comparison results of the biological function of the detected significant genes between SFMEB and the other five methods are shown in Fig. 7. SFMEB clearly finds more related genes compared with the other five methods. In particular, some important genes are only detected by SFMEB. These include ‘ENSG00000146648’, which is associated with lung cancer, ‘ENSG00000129675’, which can cause X-chromosomal non-specific cognitive disability, and mutations in ‘ENSG00000012048’, which are responsible for approximately 40% of inherited breast cancers and more than 80% of inherited breast and ovarian cancers. The biological functions of the uniquely detected significant genes between SFMEB and the other five methods are presented in Additional file 2. The results show that SFMEB provides a more accurate DE gene detection in real data of the same species.

For human and mouse RNA-seq counts data, each species has two groups of orthologous transcripts, which were obtained by using the mRNA-seq Sample Prep Kit (Illumina) platform with paired-end sequencing [10]. We apply a similar analysis process as same species and detect more DE genes by setting the reject rate at 20% for the training data, for a final detection of 4389 DE orthologous genes. Likewise, we compare the number of conserved orthologous genes that are included in the detected 4389 DE genes, as well as in the 500 and 1000 most significant genes. The results are shown in Supplemen-tary Table S3. SFMEB identifies the smallest number of known conserved orthologous genes and therefore has the smallest FDR. We then analyse the biological function of the 200 most significant genes that exclude common genes. Supplementary Figure S14 shows the number of common genes detected by at least two methods and the genes that were only detected by one method. The pairwise comparison results for the biological function of the detected significant genes between SFMEB and Median and SCBN are shown in Supplementary FigureS15. Similarly, SFMEB detects more genes related to illness or evolution, such as ‘ENSG00000131095’ that can cause Alexander disease, ‘ENSG00000128656’ that is associated with Duane’s retraction syndrome 2 (DURS2), ‘ENSG00000165795’ that is involved in glioblastoma carcinogenesis, and ‘ENSG00000197746’ that is associated with Gaucher disease and metachromatic leukodystrophy. These genes are not included in the 200 most significant genes detected by other two methods. The biological functions of the uniquely detected significant orthologous genes between SFMEB and the other two methods are presented in Additional file 3.

## Discussion

High-throughput sequencing is an advanced technology for conducting biological research; however, detecting DE genes between the same or different species is still a challenge. DE analysis tries to find a set of genes for classification or identification of the features that corresponding to the interested biological phenomenon. Although several methods have been proposed to detect DE genes in the same species, the demand for DE gene detection in different species continues to increase, including the exploration of gene evolution in mammalian organs [10] and the comparison of medicine effect of gene expression levels [30–33].

The data for the same and different species have a different structure; therefore, the existing method for the same species cannot be directly applied to different species. Furthermore, systematic variation, such as biological replicates and heterogeneity, makes the real data more complicated. We have used datasets of same species (Liver and Kidney) and different species (Human and Mouse), to illustrate one of the complex scenario in real data. We found that heterogeneity is very common in real data, which has a huge influence on the normalization methods that based on only one scaling factor. Therefore, a method to solve these problems is urgently needed.

One of the advantage of the proposed SFMEB method is that not necessary to normalize data, which is particularly attractive when the RNA-seq data include more than one scaling factor. Bying treating DE genes as outliers, SFMEB transforms the DE gene detection to an outlier detection problem. And SFMEB only uses a small set of stable genes (eg. housekeeping gene or conserved orthologous gene) to build the model. This property enables SFMEB to deal with heterogeneous data or data with biological replicates.

Simulations are further conducted to evaluate the performance of SFMEB in a broad range of possible settings. We have the following findings. First, SFMEB method outperforms the other methods in most cases, especially when the proportion of DE genes is large, the data are heterogeneous or biological replicates are present. Second, SFMEB method can set the reject rate in the training data, and thus control the type I error and ensure a lower FDR. Third, SFMEB method could be easily extended to different species without normalizaiton. The real data analysis also confirms the performance of the proposed SFMEB method in both the same species and different species. However, note that there are also some limitations of SFMEB model: firstly, it provides a dihcotomic separation of results instead of a score, such as a p-value or adjusted p-value; secondly, it depends on the choice of some parameters (such as the parameter *C* and *ν* in the model).

## Conclusions

In this paper, we propose a scaling-free minimum enclosing ball (SFMEB) method for detecting DE genes for RNA-seq data. Exploiting this SFMEB method allows ready detection of DE genes in same species as well as in different species without normalization. To satisfy implication requirement, we developed a R package MEB, with source code available at bioconductor website. The practitioners can use this method to detect DE genes based on the information of the given RNA-seq dataset and a small set of stable genes (eg. housekeeping gene or conserved orthologous gene).

The proposed method is better at detecting DE genes in some cases; however, some problems remain, such as the selection of the reject rate. In addition, the SFMEB method is insensitive when the data only include a small part of the DE genes and when biological replicates are lacking. These issues still need further consideration in our future work.

## Methods

### Notations

Let *G* be the set of all genes, and let *G*_0_ ⊂ *G* denote a small set of known non-DE genes, such as housekeeping genes. Let {*x*_*ij*_} be the observed count of short reads of gene *i* in condition *j*, where 1≤*i*≤|*G*|, and |·| represents the number of elements in a set, generally, *j*=1,2. If multiple reads are made for each condition, they can also be represented as a vector *x*_*i*_, which represents the expressions of gene *i* in all conditions. We further assume the non-DE genes are similar so that there exists a mapping, possibly nonlinear, *ϕ* that maps *x*_*i*_ into a feature space, where non-DE genes can be enclosed by a compact hypersphere. Ideally, in the feature space, all non-DE genes in *G* are contained in the ball, and those genes outside the enclosing ball can be identified as DE genes.

### Scaling-free minimum enclosing ball method

Given a set of non-DE genes *G*_0_, the scaling-free minimum enclosing ball (SFMEB) method finds a smallest ball *B*(*c*,*R*) containing all the mapped non-DE genes in the feature space, where *c* is the center of the enclosing ball and *R* is the radius. For a hard margin ball, all the non-DE genes must be contained within the ball. This can be formulated as

1 $$ \begin{array}{ll} \qquad\;\underset{\pmb{c}, R}{min}\quad R^{2} & \\ subject\;to\quad ||\phi{(\pmb{x}_{i})}-\pmb{c}||^{2}\leq R^{2}, \;for\;any\;\pmb{x}_{i} \in G_{0},&  \end{array}  $$

where *ϕ* is the implicit mapping function that maps *x*_*i*_ to the feature space.

However, in reality, enforcing the inclusion of all non-DE genes in the enclosing ball is often too restrictive and leads to a large value of *R* and, thus, a large false negative rate in terms of identifying DE genes. Therefore, a more appropriate approach is to allow some non-DE genes to lie outside the enclosing ball, as this leads to the SFMEB method with soft margin.

The key idea of a soft margin is to introduce a set of nonnegative slack variables which indicate the outliers of non-DE genes lying outside the enclosing ball. More specifically, the proposed SFMEB method with a soft margin can be formulated as

2 $$ \begin{array}{ll} \qquad\;\underset{\pmb{c}, R, \xi}{min}\quad R^{2}+C\sum_{i=1}^{n}\xi_{i}, &\\ subjuct\;to\quad ||\phi{(\pmb{x}_{i})}-\pmb{c}||^{2}\leq R^{2} +\xi_{i},\;\\ \qquad\qquad\qquad \xi_{i}\geq 0, \; i=1, 2, \cdots, n,&  \end{array}  $$

where *ξ*_*i*_ is the slack variable, and *C* is a tuning parameter controlling the ball radius and the number of errors. The Lagrangian of the primal problem (2) can be written as

3 $$ {}\begin{array}{ll} L(\pmb{c}, R, \xi, \alpha, \beta)=R^{2}+C\sum_{i=1}^{n}\xi_{i}+\sum_{i=1}^{n}\alpha_{i}(||\phi{(\pmb{x}_{i})}-\pmb{c}||^{2}\\ \qquad\qquad\qquad\quad-R^{2}-\xi_{i})-\sum_{i=1}^{n}\beta_{i}\xi_{i},  \end{array}  $$

where *α*_*i*_≥0 and *β*_*i*_≥0 are the Lagrange multipliers.

By the duality principle, the dual problem of (2) is

4 $$ \begin{array}{ll} \underset{\alpha, \beta}{max}\,\underset{\pmb{c}, R, \xi}{min}\, L(\pmb{c}, R, \xi, \alpha, \beta).  \end{array}  $$

We first calculate the minimum of *L*(*c*,*R*,*ξ*,*α*,*β*) in terms of *c*, *R* and *ξ*, and we have

5 $$ \pmb{c}=\sum_{i=1}^{n}\alpha_{i}\phi(\pmb{x}_{i}).\;  $$

Plugging formula (5) into (4), we obtain the equivalent dual form

6 $$ {}\begin{array}{ll} \qquad\;\underset{\alpha}{min}\quad \sum_{i=1}^{n}\sum_{j=1}^{n}\alpha_{i}\alpha_{j}K(\pmb{x}_{i},\pmb{x}_{j})-\sum_{i=1}^{n}\alpha_{i} K(\pmb{x}_{i},\pmb{x}_{i}) &\\ subject\;to\quad \sum_{i=1}^{n}\alpha_{i}=1, \\ \qquad\qquad\qquad 0\leq\alpha_{i}\leq C, \; i=1,2,\cdots, n.&  \end{array}  $$

where *K* is a kernel function satisfying *K*(*x*_*i*_,*x*_*j*_) = *ϕ*(*x*_*i*_)^*T*^*ϕ*(*x*_*j*_). Note that the optimization problem in (6) is a typical quadratic programming (QP) task that can be solved by many standard QP packages. Furthermore, by the Karush-Kuhn-Tucker (KKT) condition, $\hat {R^{2}}=||\phi {(\pmb {x}_{i}^{*})}-\pmb {c}||^{2}$, where $\pmb {x}_{i}^{*}$ is support vector.

By solving (6) for $\hat \alpha _{i}$, we obtain the decision function of the SFMEB method

7 $$ \begin{array}{ll} \hat{f}(\pmb{x}_{i})=||\phi{(\pmb{x}_{i})}-\hat{\pmb{c}}||^{2}-\hat{R}^{2} \\ \qquad\;\ =K(\pmb{x}_{i},\pmb{x}_{i})-2\sum_{j=1}^{n}\hat{\alpha}_{j}K(\pmb{x}_{i},\pmb{x}_{j})\\ \qquad\;\ +\sum_{i=1}^{n}\sum_{j=1}^{n}\hat{\alpha}_{i}\hat{\alpha}_{j}K(\pmb{x}_{i},\pmb{x}_{j})-\hat{R}^{2}. \end{array}  $$

The gene is regarded as a DE gene if *f*(*x*_*i*_) > 0 and as a non-DE gene otherwise.

One of the key advantages of the proposed method is that it does not require normalization of the raw data in advance, so it circumvent the difficulties of inappropriate normalization and other complex matters with more than two scaling factors. This flexibility means that the SFMEB method can be easily extended to handle the detection of DE genes in different species.

Generally, we use the radial basis function (RBF) as a kernel function, *K*(*x*_*i*_,*x*_*j*_)=*exp*(−*ν*||*x*_*i*_−*x*_*j*_||^2^), with a scale parameter *ν*. For the QP problem of (6), we have tuning parameters of *C* and *ν*. We can simplify the tuning process by fixing *C* = 0.1 and selecting *ν* by a grid search in (0,1). In order to control the scale of the ball in the feature space, we also need to set the reject rate, which represents the proportion of non-DE genes in the training data that will be regarded as DE genes. In real data, we usually set this parameter according to the complexity of data: the more complex the data, the larger the reject rate. In this paper, we set the reject rate as 10% in most cases. SupplementaryFigure S16 shows the error rates of training data and test data in a simulation data when fixing the parameter *C* as 0.01, 0.1, and 1, separately, and selecting the value of *ν* in (0,1) by the same steps. Apparently, it is more reasonable to set *C* as 0.1 to ensure the best test error when taking 10% training error (10% reject rate).

## Supplementary Information

**Additional file 1** Supplementary figures and tables. This file contains related figures and tables for simulated and real datasets.

**Additional file 2** The details for differentially expressed transcripts for same species data. The biological functions of the uniquely detected significant genes between SFMEB and the other five methods.

**Additional file 3** The details for differentially expressed transcripts for different species data. The biological functions of the uniquely detected significant genes between SFMEB and the other two methods.

## Data Availability

The liver and kidney (Marioni et al. (2008) [25]) RNA-seq dataset is available in the GEO database with accession number GSE11045, and the human and mouse (Brawand et al. (2011) [10]) RNA-seq dataset is available with accession number GSE30352. All the R scripts that analysed the data could be accessible at https://github.com/\FocusPaka/NIMEB. Additional supporting Figures and Tables are included as Additional files.

## Abbreviations

DE: Differentially expressed
SFMEB: Scaling-free minimum enclosing ball
NGS: Next-generation sequencing
RPKM: Reads per kilobase of transcript per million mapped reads
SCBN: Scale-based normalization
TMM: Trimmed mean of *M*-values
CVM: Core vector machines
RBF: Radial basis function
FDR: False discovery rate
ROC: Receiver operating characteristic
AUC: Area under the curve
