# Supplementary material for: A scaling-free minimum enclosing ball method to detect differentially expressed genes for RNA-seq data
Source: BMC Genomics. 2021 Jun 26;22:479. doi: 10.1186/s12864-021-07790-0 (PMC8234728; doi:10.1186/s12864-021-07790-0)
Supplement: Supplementary file 1 — Additional file 1 Supplementary figures and tables. This file contains related figures and tables for simulated and real datasets. [file 12864_2021_7790_MOESM1_ESM.pdf]

Supplemental materials for "A scaling-free minimum enclosing ball method to detect differentially expressed genes for RNA-seq data"

Yan Zhou [1], Bin Yang [1], Junhui Wang [2], Jiadi Zhu [1,*] and Guoliang Tian [3,*]

[1]*College of Mathematics and Statistics, Institute of Statistical Sciences, Shenzhen Key Laboratory of Advanced Machine Learning and Applications, Shenzhen University, Shenzhen, China;* [2]*School of Data Science, City University of Hong Kong, Hong Kong;* [3]*Department of Statistics and Data Science, Southern University of Science and Technology, Shenzhen, China*

Supplementary Figures (16) and Tables (3)

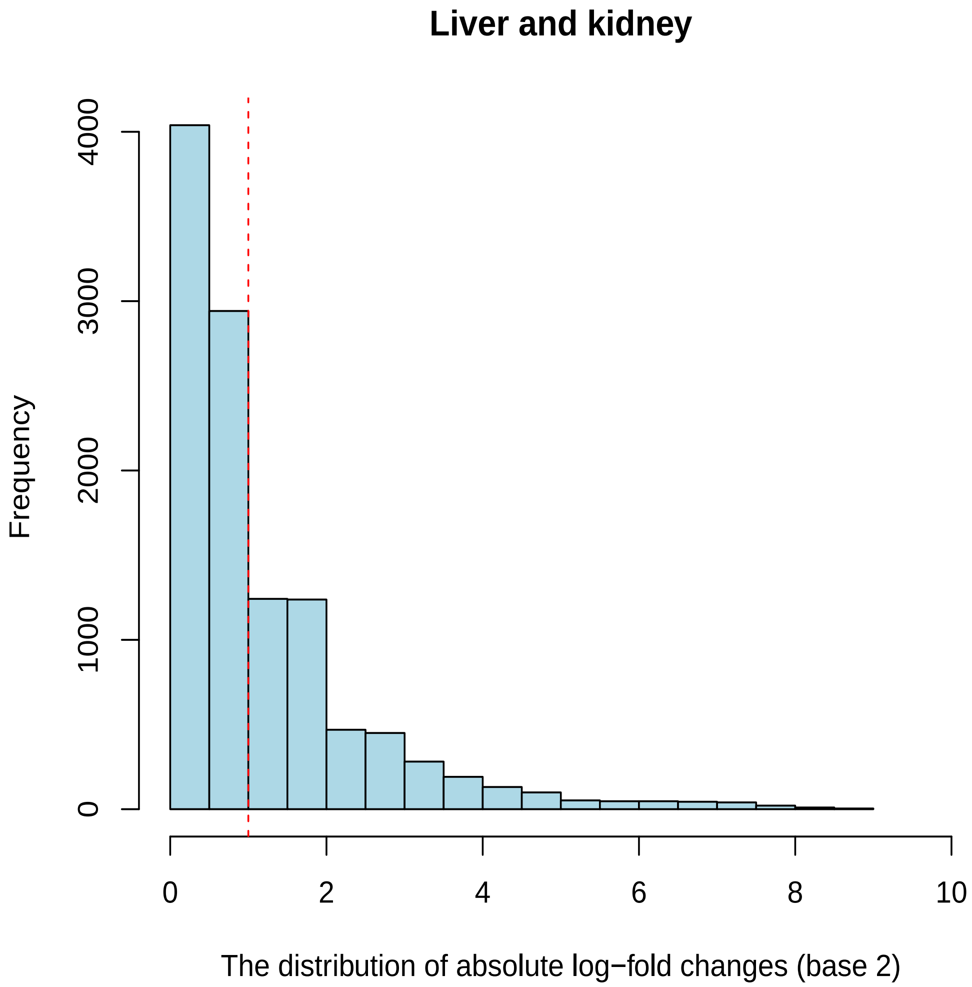

Figure S1: The distribution of log-fold changes (base 2) of the transcripts in liver and kidney (Marioni et al. [1]). The red dashed line indicates a thresholding value for discriminating DE and non-DE genes.

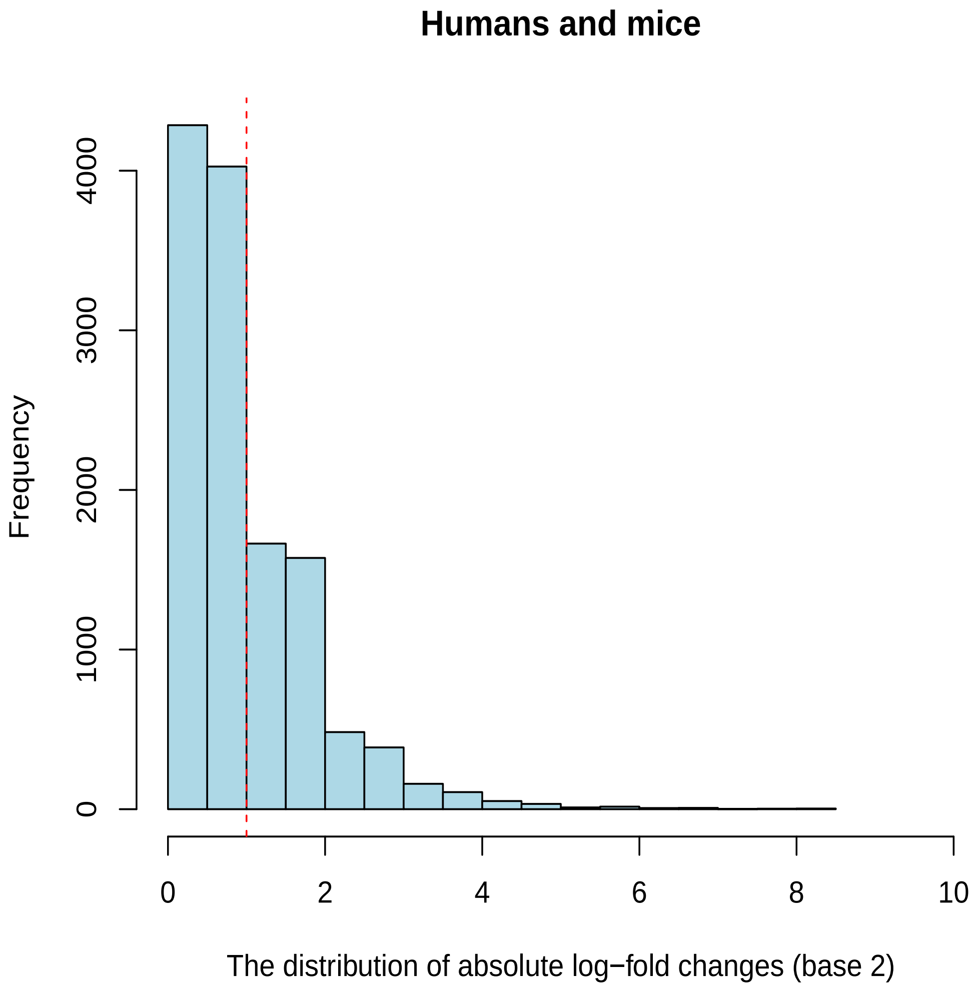

Figure S2: The distribution of log-fold changes (base 2) in transcripts between humans and mice (Brawand et al. [2]). The red dashed line indicates a thresholding value to discriminate DE and non-DE genes.

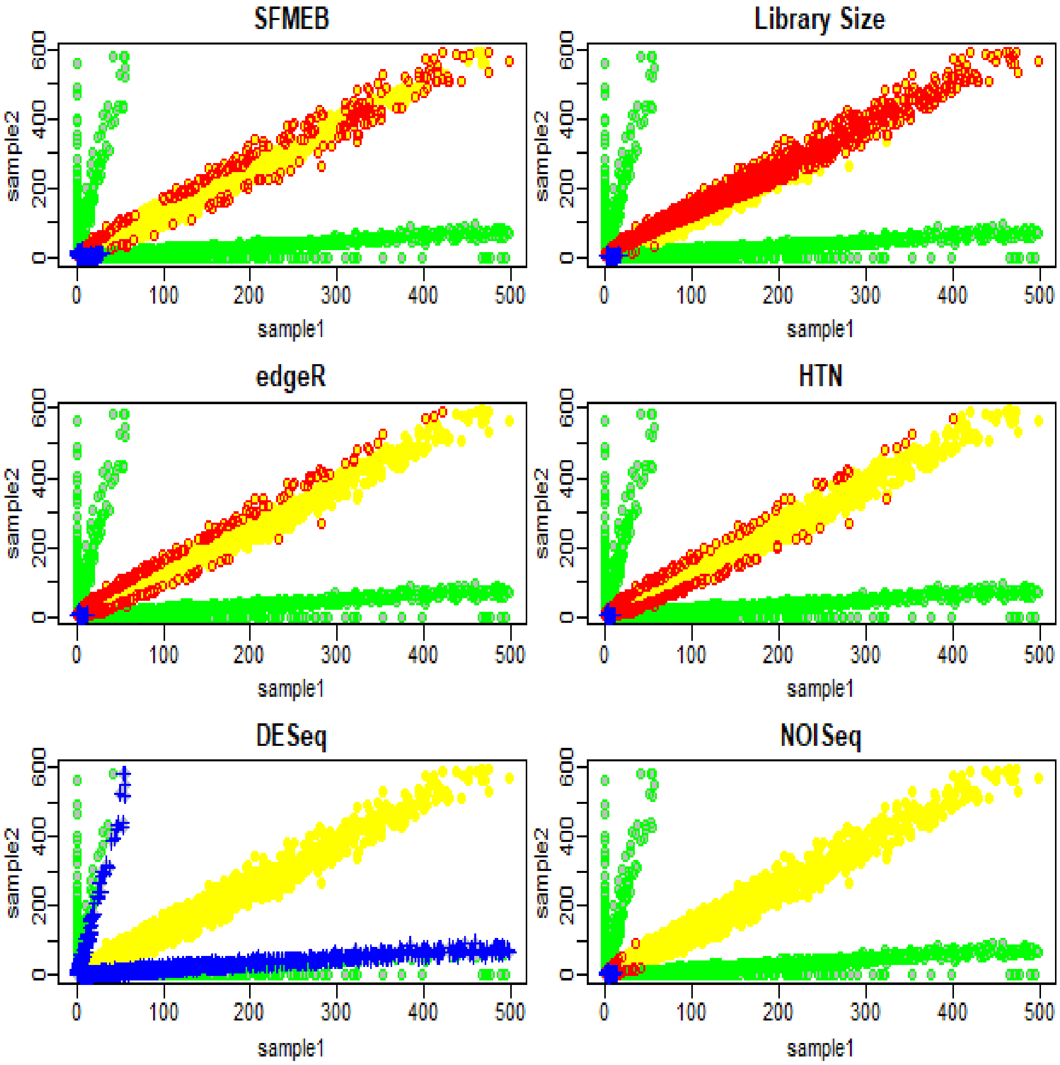

Figure S3: In Study 1, the discrimination results for the six methods when the proportion of DE genes is 0.2. Each point represents a gene, the coordinates of point are the counts of gene in two samples. The yellow points represent those genes that are non-DE and can be detected as non-DE genes. The green points represent those genes that are DE and can be detected as DE genes. The red points represent those genes that are non-DE but can be detected as DE genes. The blue points represent those genes that are DE but can be detected as non-DE genes.

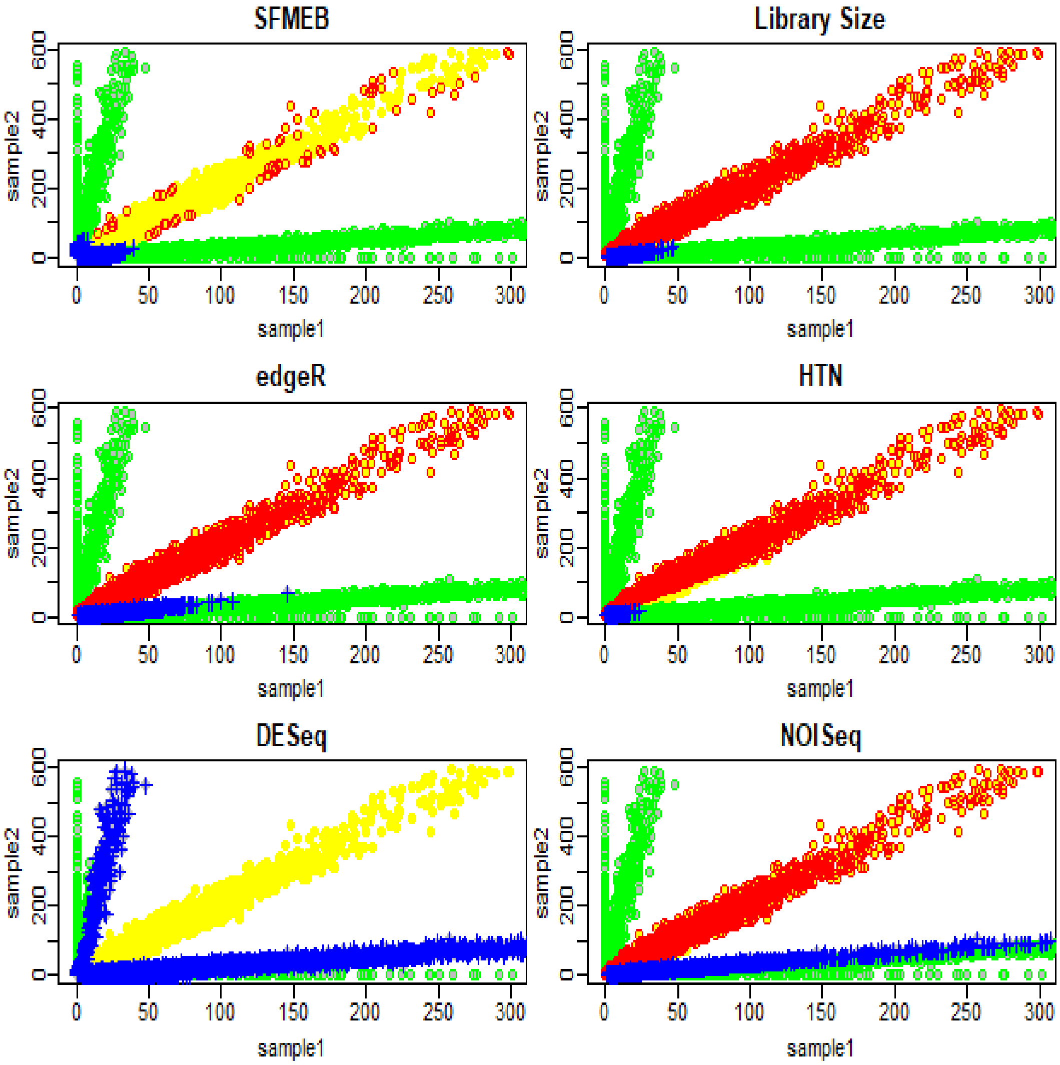

Figure S4: In Study 1, the discrimination results for the six methods when the proportion of DE genes is 0.6. Each point represents a gene, the coordinates of point are the counts of gene in two samples. The yellow points represent those genes that are non-DE and can be detected as non-DE genes. The green points represent those genes that are DE and can be detected as DE genes. The red points represent those genes that are non-DE but can be detected as DE genes. The blue points represent those genes that are DE but can be detected as non-DE genes.

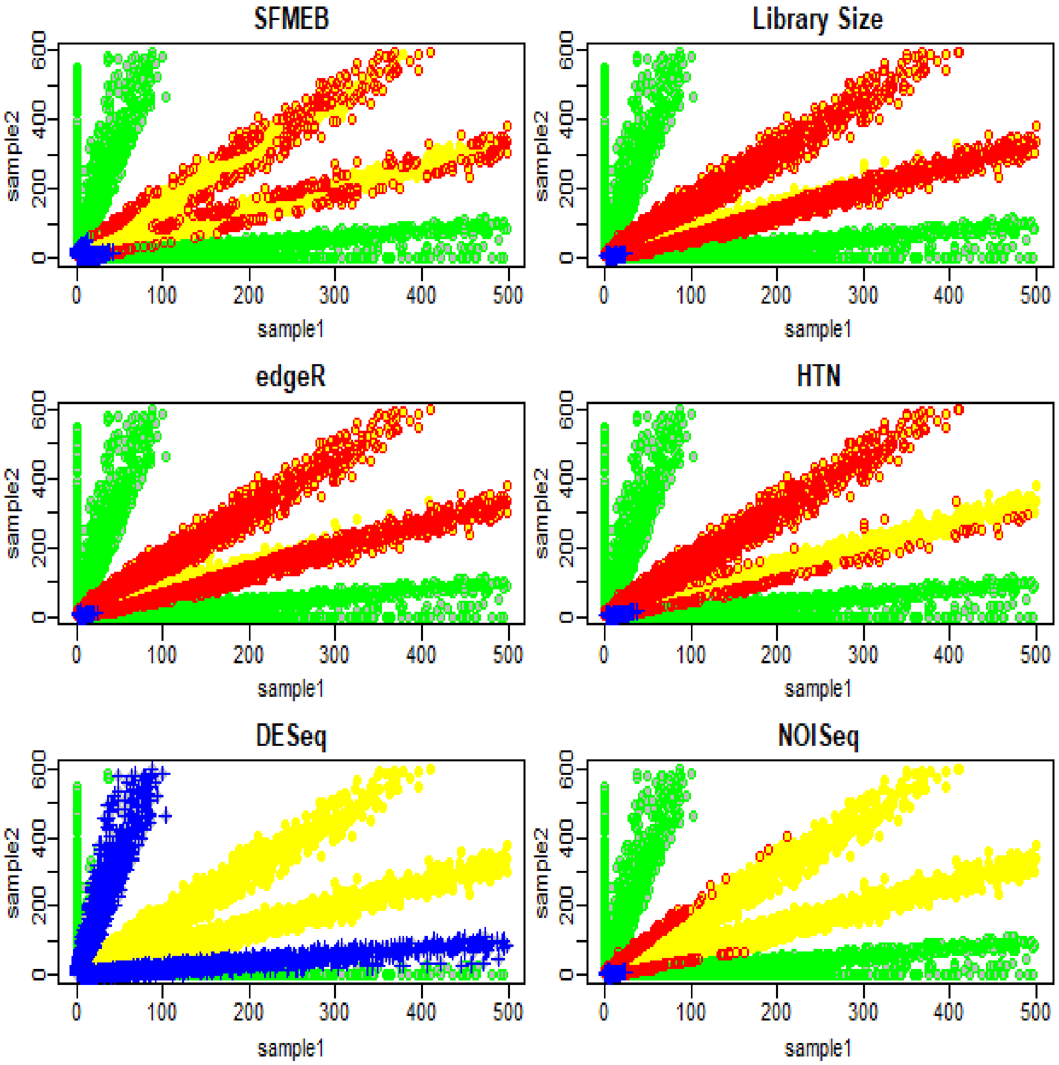

Figure S5: In Study 2, the data are heterogeneous, with two scaling factors, and the proportion of DE genes in the first dataset is fixed at 0.3. The discrimination results are shown for the six methods when the proportion of DE genes in the second dataset is equal to 0.2. Each point represents a gene, the coordinates of point are the counts of gene in two samples. The yellow points represent those genes that are non-DE and can be detected as non-DE genes. The green points represent those genes that are DE and can be detected as DE genes. The red points represent those genes that are non-DE but can be detected as DE genes. The blue points represent those genes that are DE but can be detected as non-DE genes.

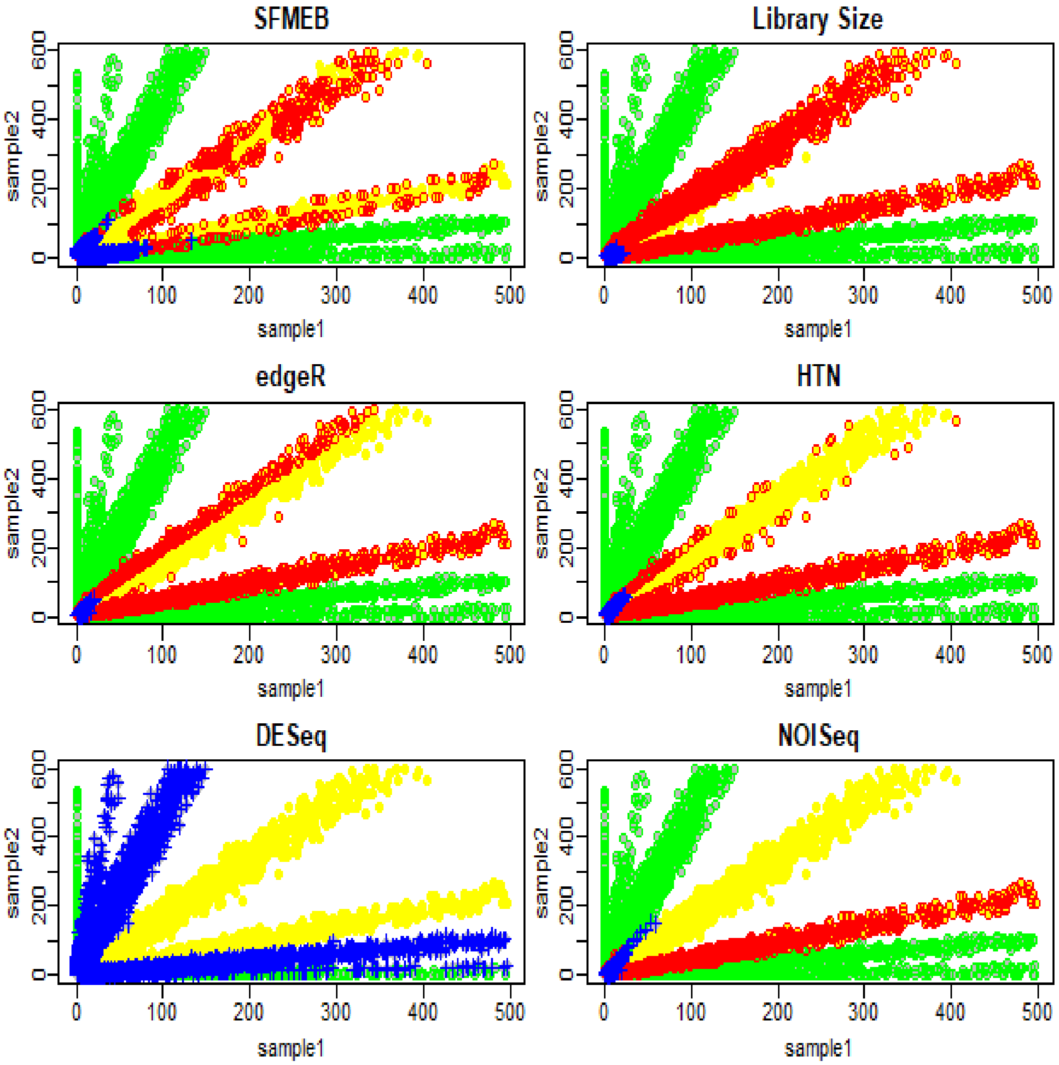

Figure S6: In Study 2, the data are heterogeneous, with two scaling factors, and the proportion of DE genes in the first dataset is fixed at 0.3. The discrimination results are shown for the six methods when the proportion of DE genes in the second dataset is equal to 0.6. Each point represents a gene, the coordinates of point are the counts of gene in two samples. The yellow points represent those genes that are non-DE and can be detected as non-DE genes. The green points represent those genes that are DE and can be detected as DE genes. The red points represent those genes that are non-DE but can be detected as DE genes. The blue points represent those genes that are DE but can be detected as non-DE genes.

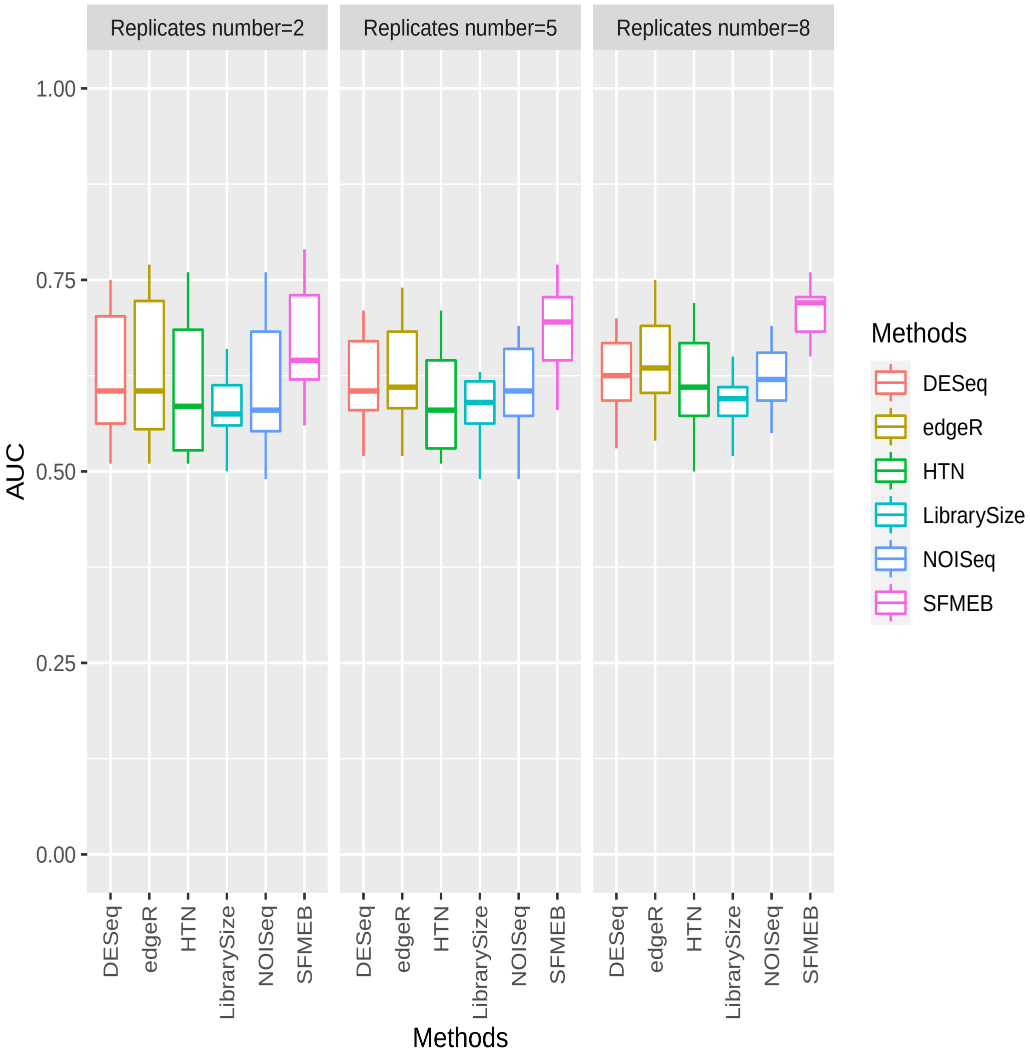

Figure S7: In Study 6, the simulation data are heterogeneous, with two scaling factors, and with biological replicates. Simulation data are generated by an RNA-seq simulator (compcodeR::generateSyntheticData(), Soneson et al. (2013)). The AUC values of the six methods are shown under the different number of biological replicates.

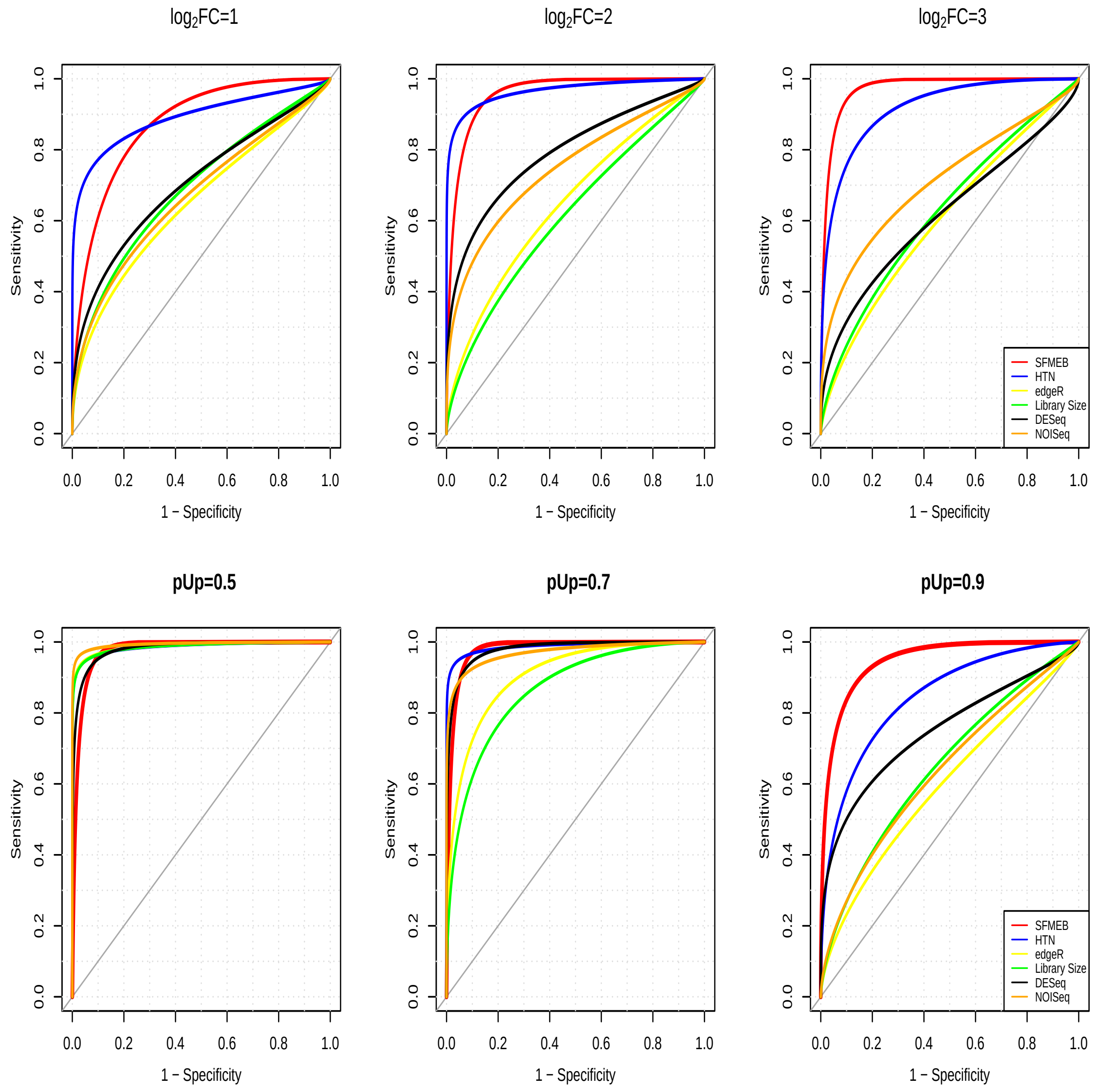

Figure S8: In Study 1, the simulation data are non-heterogeneous without replicates, and are generated by following the steps of Robinson and Oshlack (2010). The Receiver Operating Characteristic (ROC) curves for six methods when the value of logFC equals to 1, 2, and 3 (the first panel). And the ROC curves for six methods when the proportion of up-regulated in all DE genes of simulation data equals to 0.5, 0.7, and 0.9 (the second panel).

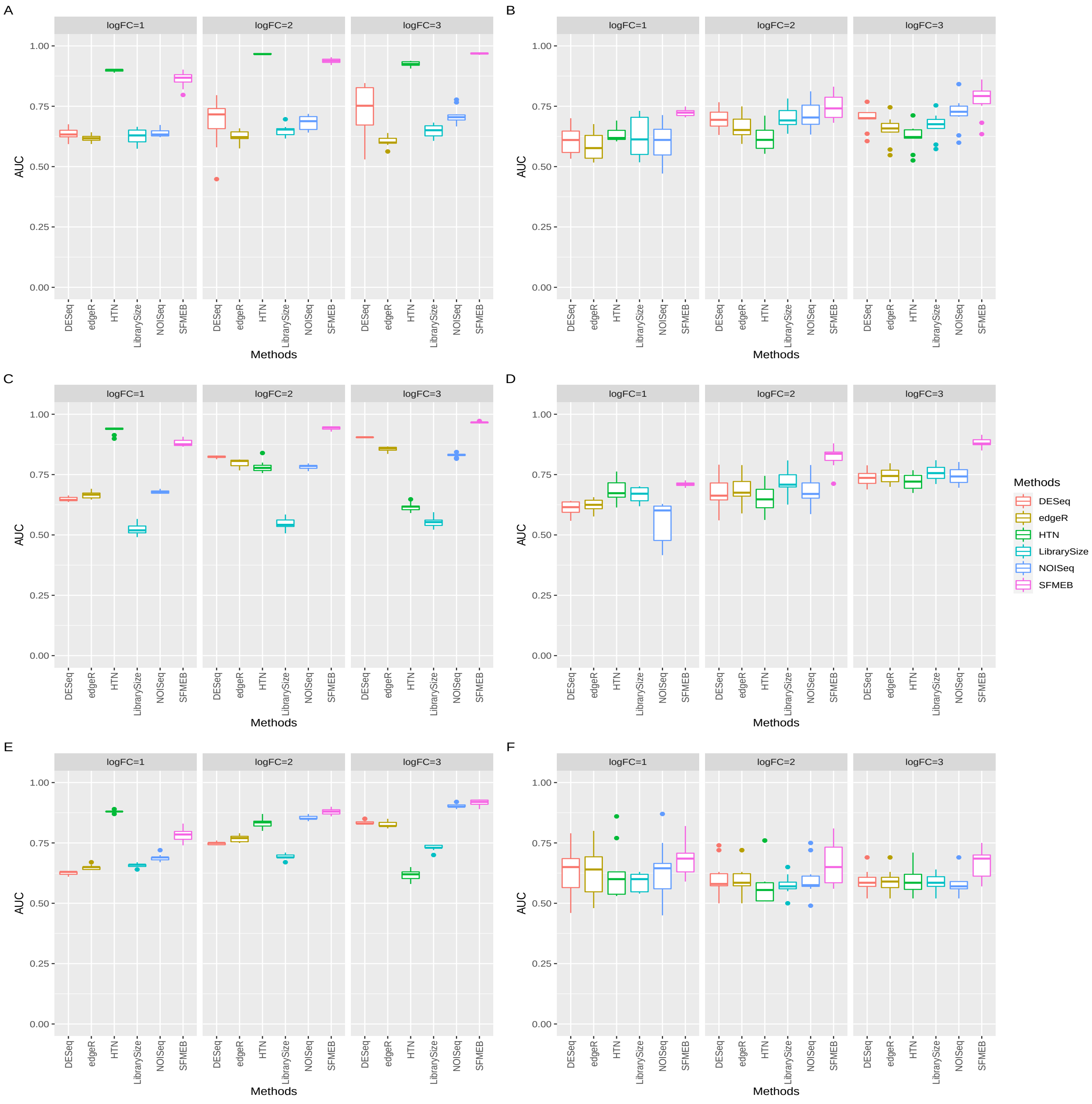

Figure S9: In six studies, the AUC boxplots of six methods with different setting of logFC (base 2) values. Figures A to F are corresponding to the six studies in the simulation studies. The data in Figures A to D are generated by a Poisson distribution [3], and the data in Figures E and F are generated by a Negative Binomial distribution [4]. Since the simulation data in Figures E and F with varied logFCs, we only tagged the lower bound of logFCs on the top of images.

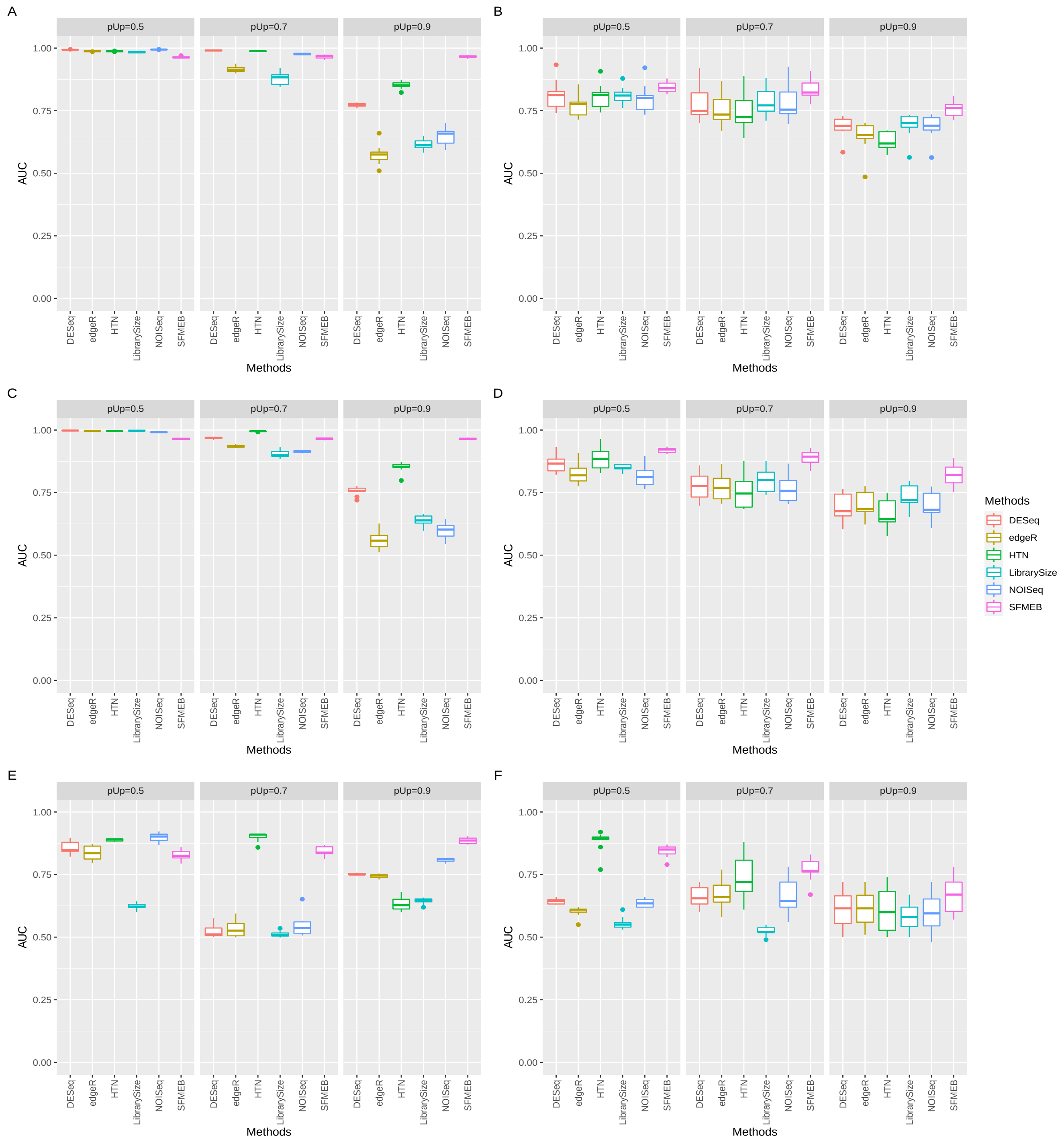

Figure S10: The AUC boxplots of six methods by changing the proportion of up-regulated genes in one condition in the six studies. Figures A to F are corresponding to the six studies in the simulation studies. The data in Figures A, C, and E are generated with one scaling factor, and the data in Figures B, D, and F are generated with two scaling factors.

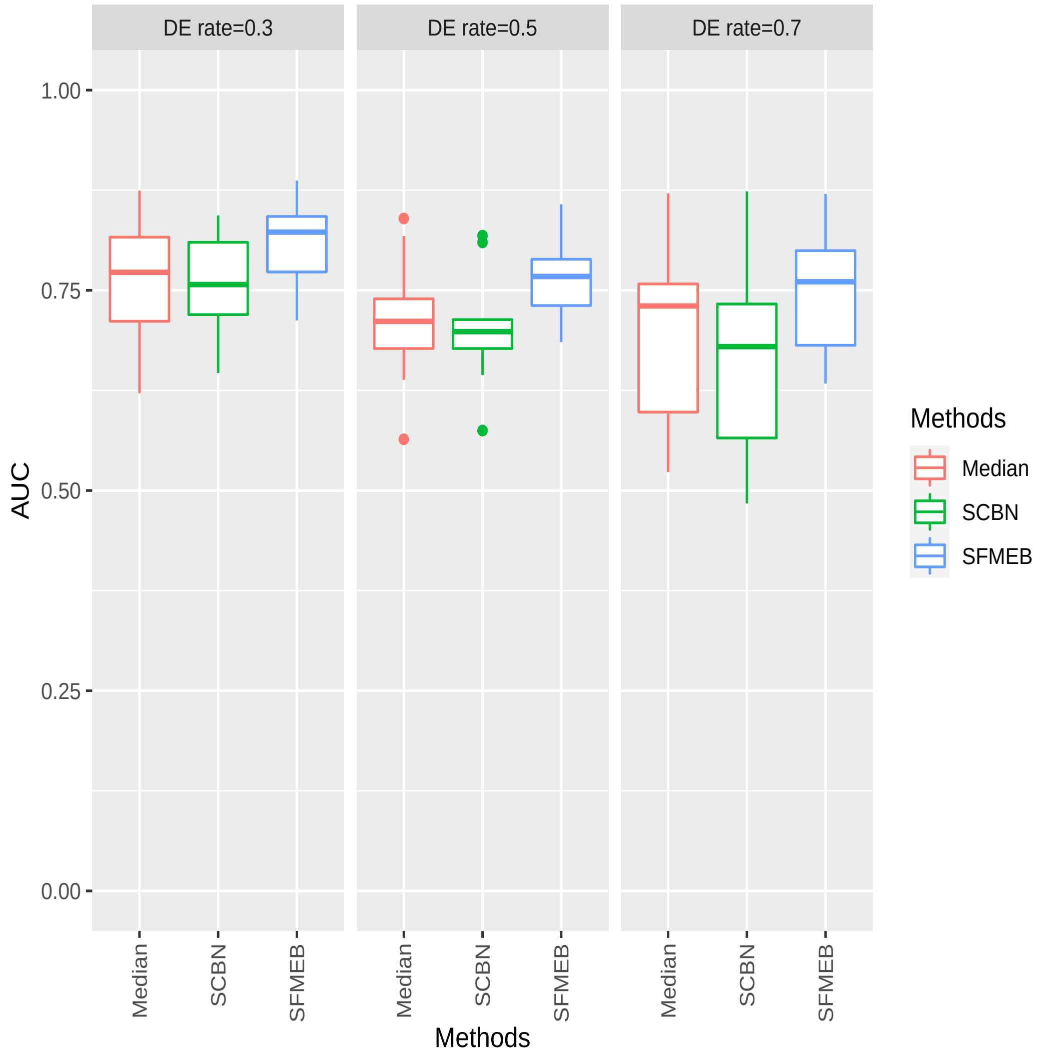

Figure S11: The AUC values for different species for the three methods when the data are heterogeneous, with two scaling factors. The proportion of DE orthologous genes in the first data group is 0.4, and the DE proportion in the second data group is equal to 0.3, 0.5, and 0.7.

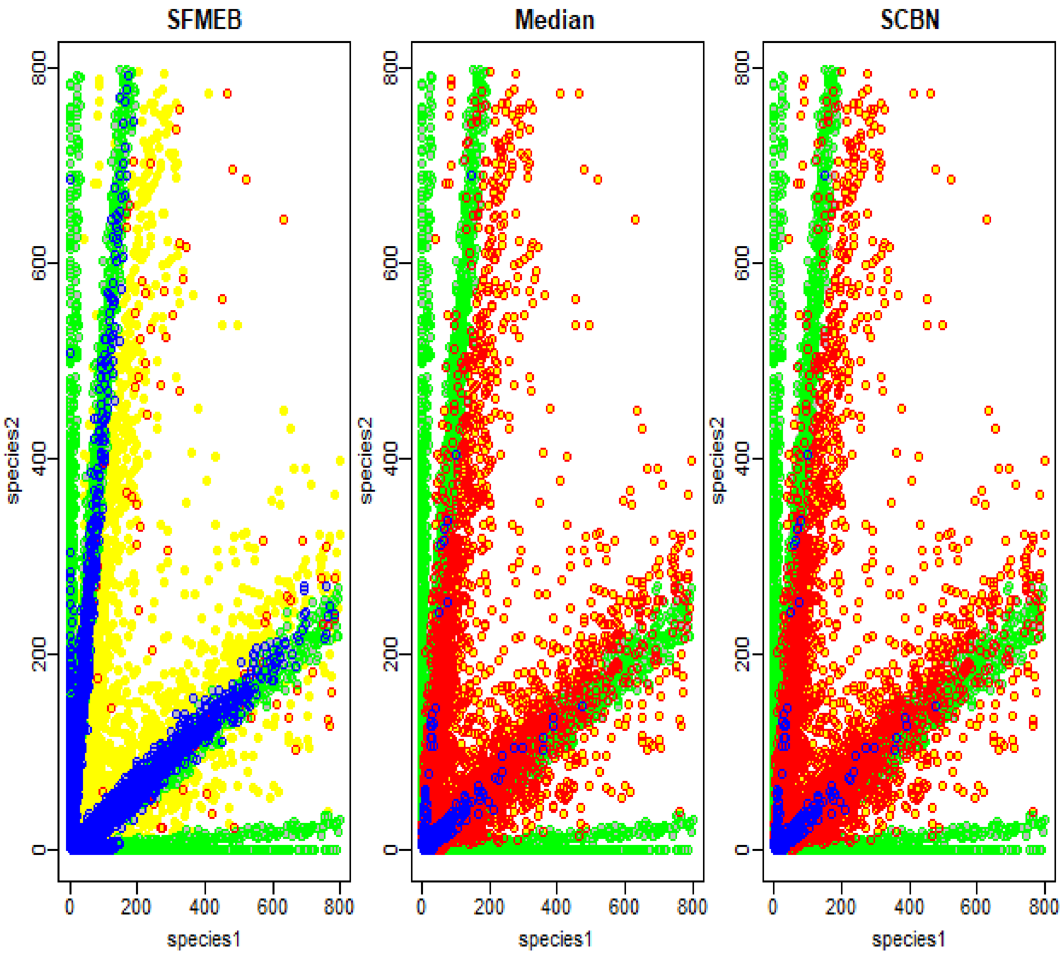

Figure S12: For different species, the data are heterogeneous, with two scaling factors. The discrimination results of the DE orthologous genes are shown for three methods when the proportion of DE orthologous genes is equal to 0.6. Each point represents a gene, the coordinates of point are the counts of gene in two samples. The yellow points represent those genes that are non-DE and can be detected as non-DE genes. The green points represent those genes that are DE and can be detected as DE genes. The red points represent those genes that are non-DE but can be detected as DE genes. The blue points represent those genes that are DE but can be detected as non-DE genes.

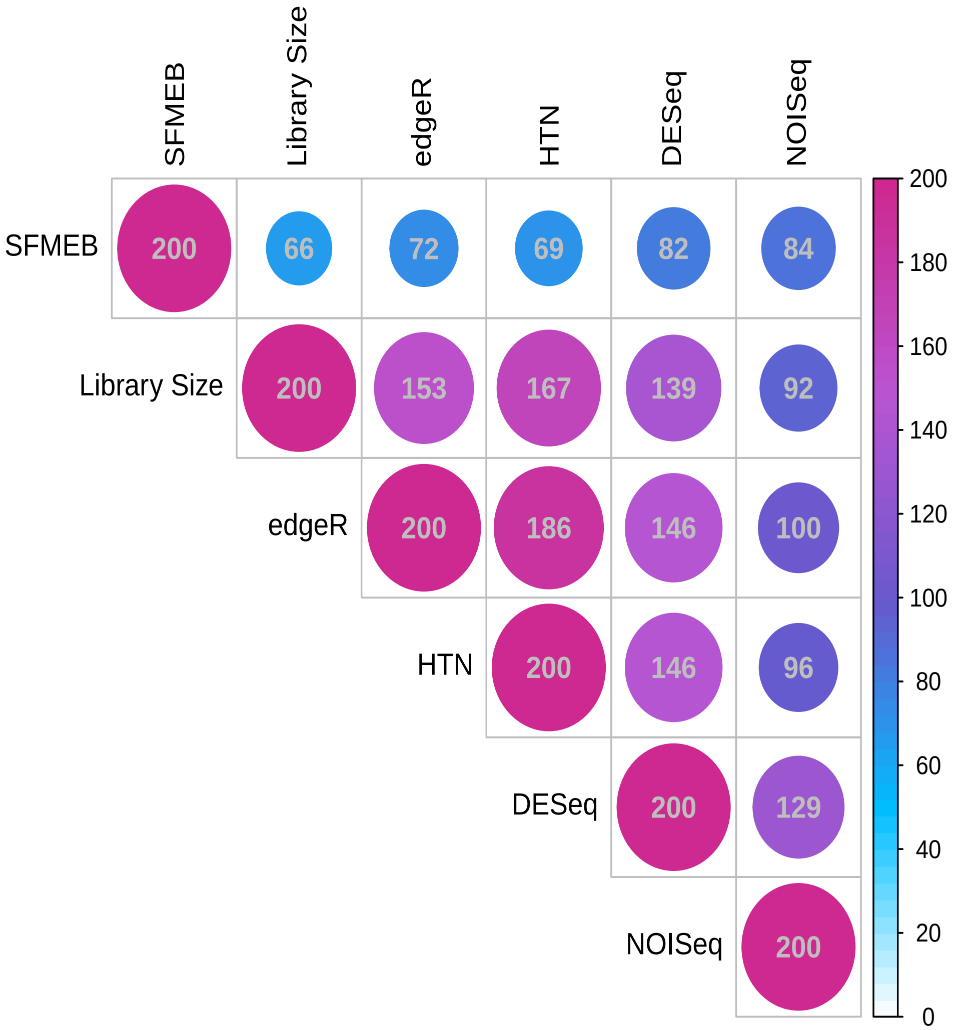

Figure S13: The common gene numbers detected by each pair of six methods in the most significant 200 genes in the liver and kidney dataset.

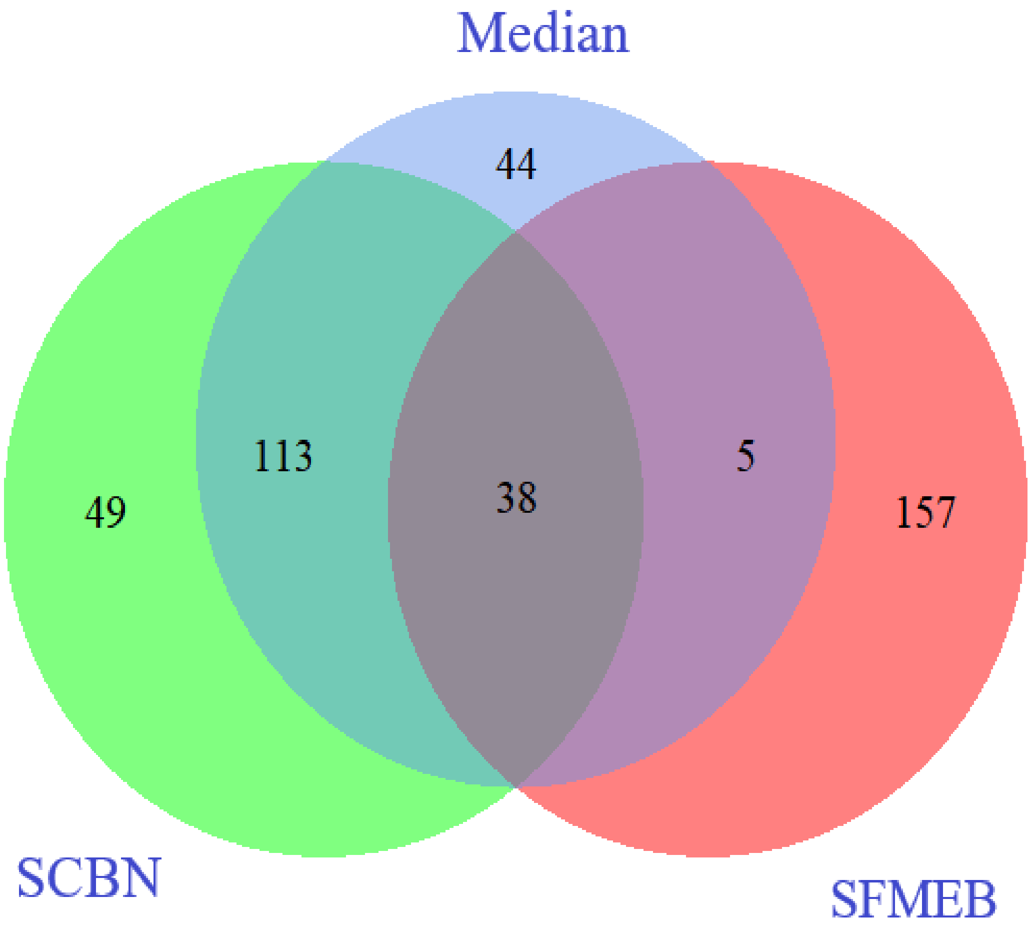

Figure S14: The common and the unique DE genes detected by each method in the most significant 200 genes in the human and mouse dataset.

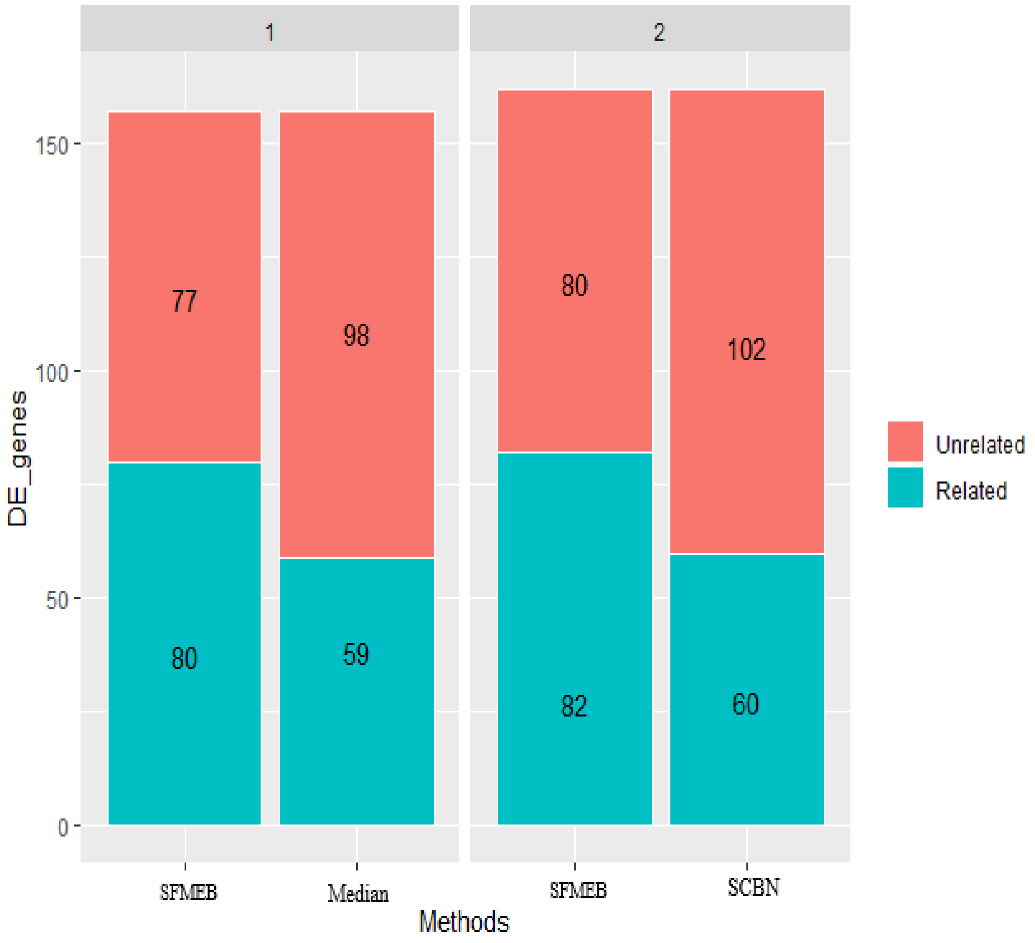

Figure S15: The pairwise comparison between SFMEB, Median, and SCBN for the most significant 200 orthologous genes, excluding common genes, in different species. The barplots show the number of genes that are related to illness and evolution (Related_LK) or Unrelated.

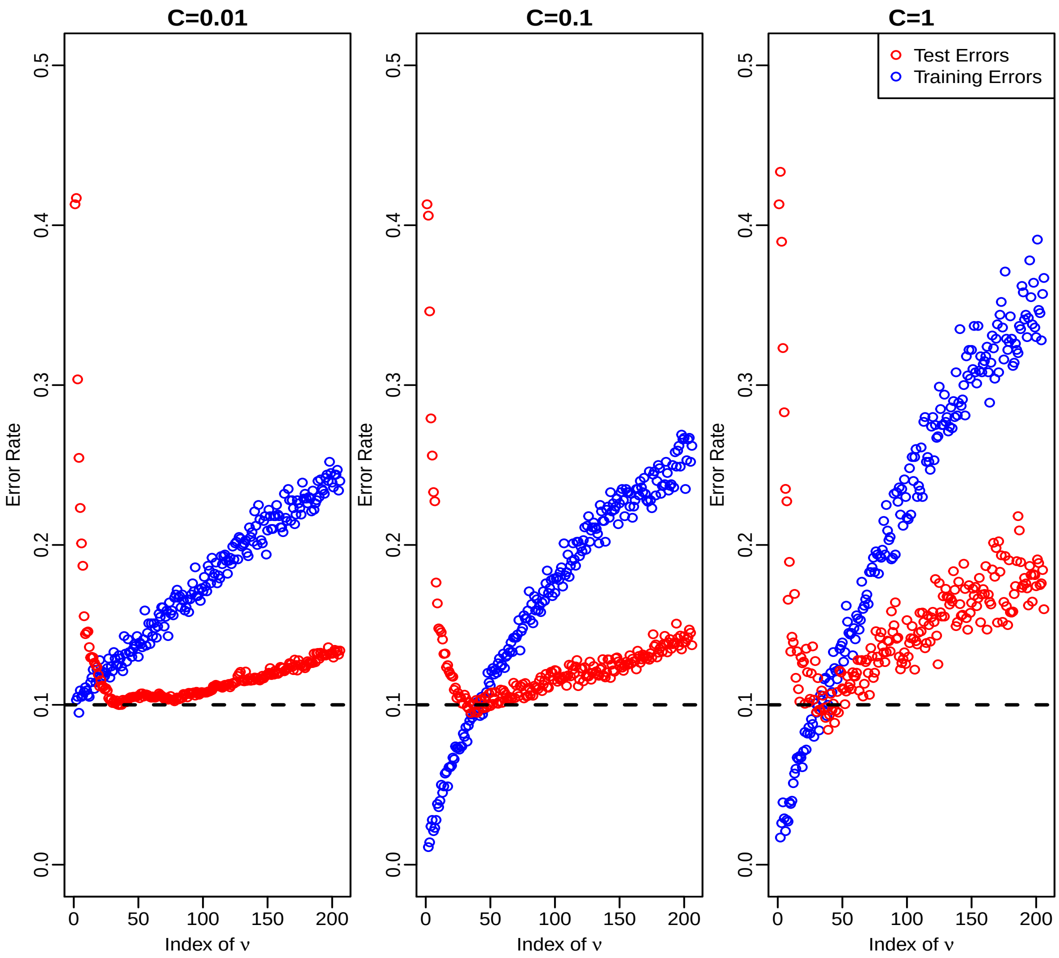

Figure S16: The error rates of training data and test data in a simulation data when fixing the parameter C as 0.01, 0.1, and 1, separately, and selecting the value of ν in $(0, 1)$ by the same steps. The x-axis is the index of ν in the $(0, 1)$.

Table S1: The number of possibly uniquely expressed genes in the two real datasets.

The same species	Total	Unique
Kidney	16519	508
Liver	16519	1099
The different species		
Human	19330	833
Mouse	19330	987

Table S2: The CPU time (seconds) required to run the differential expression analysis for each of the eavluated methods, for different sample sizes.

Number of samples	1	3	5
SFMBE	0.93	1.44	2.04
HTN	90.00	455.94	814.84
edgeR	0.54	2.71	2.98
Library Size	0.53	63.13	63.32
DESeq	3.40	5.41	5.01
NOISeq	35.57	27.23	26.02

Table S3: For different species, the number of detected conserved orthologous genes in the most significant 500, 1000, and 4389 DE genes for the three methods.

Number of DE genes	500	1000	4389
SFMEB	0	8	13
Median	13	22	72
SCBN	12	22	68

References

[1] Marioni, J.C., Mason, C.E., Mane, S.M., Stephens, M., Gilad, Y.: RNA-seq: an assessment of technical reproducibility and comparison with gene expression arrays. Genome Research **18(9)**, 1509–1517 (2008)

[2] Brawand, D., Soumillon, M., Necsulea, A., Julien, P., Csrdi, G., Harrigan, P., Weier, M., Liechti, A., Aximu-Petri, A., Kircher, M., Albert, F.W., Zeller, U., Khaitovich, P., Grtzner, F., Bergmann, S., Nielsen, R., Pbo, S., Kaessmann, H.: The evolution of gene expression levels in mammalian organs. Nature **478**, 343–348 (2011)

[3] Robinson, M.D., Oshlack, A.: A scaling normalization method for differential expression analysis of RNA-seq data. Genome Biology **11**, 25 (2010)

[4] Soneson, C., Delorenzi, M.: A comparison of methods for differential expression analysis of RNA-seq data. BMC Bioinformatics **14**, 91 (2013)
